# ASTWAS: modeling alternative polyadenylation and SNP effects in kernel-driven TWAS reveal novel genetic associations for complex traits

**DOI:** 10.1093/bib/bbaf725

**Published:** 2026-01-19

**Authors:** Yan Wang, Lei Wang, Nan Sheng, Jie Hong, Yunzhi Liu, Pengze Wu, XinFei Wang, Shuyan Zhang, Chen Cao

**Affiliations:** Key laboratory of Symbolic Computation and Knowledge Engineering of Ministry of Education, College of Computer Science and Technology, Jilin University, No. 2699 Qianjin Street, Chaoyang District, 130012 Changchun, Jilin, China; Key laboratory of Symbolic Computation and Knowledge Engineering of Ministry of Education, College of Computer Science and Technology, Jilin University, No. 2699 Qianjin Street, Chaoyang District, 130012 Changchun, Jilin, China; Shenzhen Loop Area Institute, No. 6 Hongmian Road, Futian Bonded Zone, Futian District, 518045 Shenzhen, Guangdong, China; Key laboratory of Symbolic Computation and Knowledge Engineering of Ministry of Education, College of Computer Science and Technology, Jilin University, No. 2699 Qianjin Street, Chaoyang District, 130012 Changchun, Jilin, China; Key laboratory of Symbolic Computation and Knowledge Engineering of Ministry of Education, College of Computer Science and Technology, Jilin University, No. 2699 Qianjin Street, Chaoyang District, 130012 Changchun, Jilin, China; Key laboratory of Symbolic Computation and Knowledge Engineering of Ministry of Education, College of Computer Science and Technology, Jilin University, No. 2699 Qianjin Street, Chaoyang District, 130012 Changchun, Jilin, China; Jiangsu Key Laboratory for Biomedical Electromagnetic Precision Theranostics, School of Biomedical Engineering and Informatics, Nanjing Medical University, No. 101 Longmian Avenue, Jiangning District, 211166 Nanjing, Jiangsu, China; Key laboratory of Symbolic Computation and Knowledge Engineering of Ministry of Education, College of Computer Science and Technology, Jilin University, No. 2699 Qianjin Street, Chaoyang District, 130012 Changchun, Jilin, China; National Key Laboratory of Intelligent Tracking and Forecasting for Infectious Diseases, Beijing Ditan Hospital, Capital Medical University, No. 8 Jingshun East Street, Chaoyang District, 100015 Beijing, China; Beijing Institute of Infectious Diseases, No. 8 Jingshun East Street, Chaoyang District, 100015 Beijing, China; Jiangsu Key Laboratory for Biomedical Electromagnetic Precision Theranostics, School of Biomedical Engineering and Informatics, Nanjing Medical University, No. 101 Longmian Avenue, Jiangning District, 211166 Nanjing, Jiangsu, China

**Keywords:** alternative polyadenylation (APA), transcriptome-wide association studies (TWAS), kernel methods, nonlinear genetic effects, complex trait susceptibility

## Abstract

Alternative polyadenylation (APA) of $3^{\prime}$untranslated regions ($3^{\prime}$UTRs) is a pervasive mechanism that regulates mRNA stability, localization, and translational efficiency by generating isoforms with distinct $3^{\prime}$UTR lengths and regulatory element composition. Despite its critical role in fine-tuning gene expression, APA has been largely overlooked in transcriptome-wide association studies (TWAS), which traditionally rely on linear models of SNP effects. To bridge this gap, we developed ASTWAS, a two-stage framework that first trains APA usage prediction models (BLUP, Elastic Net, LASSO, and TOP1) to quantify SNP impacts on distal poly(A) site choice via the percentage of distal poly(A) site usage index, and then aggregates weighted SNP effects within a kernel method to capture both linear and nonlinear genetic interactions. In extensive simulations spanning additive, epistatic, heterogeneous, compensatory, and single-variant architectures under both pleiotropy and causality scenarios, ASTWAS shows higher statistical power than linear APA-TWAS ($3^{\prime}$aTWAS), especially at low heritability and in the presence of SNP interactions. Applied to WTCCC type 1 diabetes and rheumatoid arthritis cohorts, ASTWAS not only rediscovers known susceptibility genes but also suggests novel candidates (e.g. *GABBR1*, *RGL2*) that form coherent interaction modules and enrich immune-related pathways, underscoring the biological significance of our algorithm in complex trait genetics. ASTWAS is implemented in Python and freely available at https://github.com/wl-Simplecss/ASTWAS.

## Introduction

In the wake of the Human Genome Project, the focus has shifted from sequencing to elucidating how genetic variation shapes phenotypes [1]. Transcriptome-wide association studies (TWAS) have emerged as a powerful approach for linking genotype to complex traits by integrating expression quantitative trait loci (eQTL) models with GWAS data [2–7]. Since its inception by Gamazon *et al.* in 2015, TWAS has identified susceptibility genes across diverse traits, from cancer and Alzheimer’s disease to metabolic traits like height and body mass, and continues to evolve as researchers incorporate increasingly rich biological priors and advanced statistical strategies [2, 8–11]. For example, sTF-TWAS integrates transcription factor binding information to refine variant selection [12], METRO leverages multi-ethnic reference panels for cross-population robustness [13], OTTERS applies polygenic risk scoring to derive eQTL weights [14], and SR-TWAS employs ensemble machine-learning across multiple tissues [15]. However, most TWAS frameworks still rely on linear models to aggregate variant effects, limiting their ability to detect nonlinear interactions. To address this, kTWAS and vc-TWAS couple TWAS’s eQTL-based variant preselection with kernel-machine aggregation, capturing complex genetic architectures, and outperforming standard TWAS alone in simulations and real-world data [16, 17].

Alternative polyadenylation (APA) in the $3^{\prime}$ untranslated region ($3^{\prime}$UTR) is a prevalent post-transcriptional regulatory mechanism in humans [18, 19]. This process utilizes distinct poly(A) sites to generate mRNA isoforms with variable $3^{\prime}$ UTR lengths. The $3^{\prime}$ UTR harbors numerous regulatory elements, such as binding sites for microRNAs (miRNAs) and RNA-binding proteins (RBPs), which modulate the abundance and localization of mRNA and protein products [20, 21]. Consequently, alterations in APA can affect target gene translation, protein localization, and protein–protein interactions, independent of mRNA expression levels or splicing [21, 22]. Therefore, the diversity generated by APA can significantly influence gene expression and disease progression [23–25]. For instance, a single-nucleotide polymorphism (SNP; rs10954213) in the $3^{\prime}$ UTR of Interferon Regulatory Factor 5 (IRF5), which alters the length and stability of this region, has been linked to susceptibility to Systemic Lupus Erythematosus [26]. Similarly, the SNP rs78378222 in the $3^{\prime}$ UTR of the TP53 gene impairs the $3^{\prime}$-end processing of TP53 mRNA, which in turn modifies susceptibility to various cancers, including cutaneous basal cell carcinoma, prostate cancer, glioma, and colorectal adenoma [27].

Despite its pervasive role in shaping gene expression programs and its implication in disease, APA has been underutilized in transcriptome-wide association studies (TWAS). To address this gap, Cui *et al.* [28] recently introduced the $3^{\prime}$aTWAS framework, which integrates APA quantitative trait loci with GWAS data across eleven neurological disorders. Their analysis of thousands of RNA-seq samples identified hundreds of trait-associated genes, over half of which were missed by conventional TWAS, suggesting that inclusion of APA dynamics can substantially broaden the discovery of susceptibility loci. However, $3^{\prime}$aTWAS relies on a linear weighting model (FUSION) to relate SNPs to trait effects, potentially overlooking nonlinear variant interactions that contribute to complex trait architecture.

We present ASTWAS, a transcriptome-wide association framework that integrates APA of $3^{\prime}$UTRs with kernel-based aggregation of SNP effects to improve gene-trait mapping. In its first phase, ASTWAS trains APA usage models using BLUP, elastic net, LASSO, and TOP1 to assign weights reflecting each variant’s impact on $3^{\prime}$UTR choice. In the second phase, these weights are combined in a weighted linear kernel and tested by the SKAT score statistic to capture both additive and nonadditive genetic contributions. In extensive simulations, ASTWAS generally showed better performance than linear APA-TWAS ($3^{\prime}$aTWAS) across diverse genetic architectures. When applied to type 1 diabetes (T1D) and rheumatoid arthritis (RA) cohorts, ASTWAS not only rediscovers known susceptibility genes but also uncovers novel candidates whose functional networks and pathway enrichments highlight immune-related mechanisms.

## Materials and methods

### Mathematical details of ASTWAS and $3^{\prime}$aTWAS

The ASTWAS model and $3^{\prime}$aTWAS use genotype data and APA data as inputs to calculate the effect of gene variants on transcript APA during transcription, and then perform association analysis with the target trait to test whether there is an association between genes and traits [28].

First, similar to standard TWAS methods, the ASTWAS and $3^{\prime}$aTWAS models use genotype data and APA data from transcript $3^{\prime}$UTR regions to train linear prediction models [8, 28]. The model’s weight parameters represent the influence of genetic variation on APA in the transcript $3^{\prime}$UTR region. We quantify the APA information in the $3^{\prime}$UTR region of transcripts as the percentage of the distal poly(A) site usage index (PDUI) [29]. The above training process can be defined as:


(1) \begin{align*}& \mathit{\mathrm{PDUI}} = \sum_{i=1}^{n} W_{i} G_{i} + \varepsilon\end{align*}

**Figure 1:**
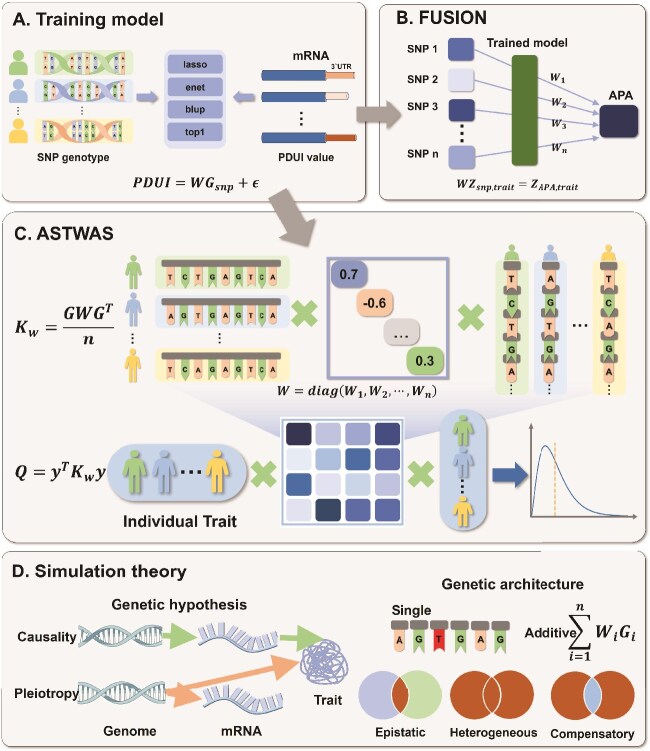
Overview of the ASTWAS model and TWAS model. (A) Training stage: Using enet, lasso, blup, and top1 models, train the PDUI value prediction model for genes using genotype data and APA data with five-fold cross-validation. (B) TWAS model: $3^{\prime}$aTWAS uses the TWAS analysis software FUSION to complete the association analysis calculation process. (C) ASTWAS Model: compared with representative TWAS methods, the ASTWAS model employs kernel machine to estimate gene genetic effects, demonstrating stronger capture of nonlinear genetic effects. (D) Simulation Methods: the robustness and effectiveness of the ASTWAS model and representative TWAS methods were primarily evaluated using simulated data incorporating various genetic structures.

In the formula, $n$ represents the number of variant sites, $G = (G_{1}, G_{2}, \cdots , G_{N})$ represents the genotype matrix, and PDUI denotes the APA data for the $3^{\prime}$UTR region of the gene transcript. $W_{i}$ is the regression parameter to be obtained during training, representing the weight of genetic variant sites on the APA of the $3^{\prime}$UTR region. To train the weight coefficients between variation and APA, the ASTWAS model and $3^{\prime}$aTWAS model both use the four statistical models provided by the FUSION software, namely BLUP, Elastic Net, LASSO, and TOP1, for five-fold cross-validation training [28] (Fig. 1A).

Next, the $3^{\prime}$aTWAS model continues to use the FUSION software to calculate the association between traits and predicted APA data [28]. For each gene, we select the model with the best prediction performance from the four trained models, obtain the weight coefficients $W$, and perform association analysis using the following equation:


(2) \begin{align*}& Z_{\mathrm{(APA,trait)}} \equiv WZ_{\mathrm{(snp,trait)}}\end{align*}

In the formula, $Z_{\mathrm{(snp,trait)}}$ is the Z-score vector of SNPs obtained from GWAS summary data through association analysis between SNPs and traits. $Z_{\mathrm{(APA,trait)}}$ represents the Z-score vector of predicted APA and phenotypes. Finally, the association between genes and target traits is determined based on $Z_{\mathrm{(APA,trait)}}$ [30, 31] (Fig. 1B).

The $3^{\prime}$aTWAS model assumes that the effects of SNPs on traits are linearly additive. Therefore, this model may not fully capture nonlinear SNP effects (including potential interactions or other nonadditive relationships). The ASTWAS model proposed in this paper uses kernel methods to capture nonlinear SNP interactions. We chose SKAT as the kernel method for the association analysis stage of the ASTWAS model to summarize the effects of genetic variation on traits [17, 32]. The SKAT method uses the Q-score test [33]. The specific formula is as follows:


(3) \begin{align*}& Q = y^{T} K y\end{align*}

In the formula, $y$ represents the phenotype vector, $K$ is the kernel function calculated from the genotype matrix $G$, $G$ is the genotype matrix, and $G_{\mathrm{ij}}$ represents the information of the $j$th gene variant locus of the $i$th individual in the genotype matrix. In this paper, a linear kernel is used to simply represent the structure of the function, which can be expressed as $k=GW_{M} G^{T}$. $W_{M}$ is the diagonal weight matrix of the minor allele frequencies (MAFs) of each variant within the target gene. Wu *et al.* [33] proposed subtracting the effects of covariates such as sex and age $(y-u)$ from the phenotype vectors, but to simplify the comparison and calculation process, this paper does not include any covariates when evaluating the model.

The ASTWAS model combines the advantages of the SKAT and $3^{\prime}$aTWAS models. The structure of the ASTWAS model is shown in Fig. 1C. In the training phase, the ASTWAS model uses the four statistical models BLUP, Elastic Net, LASSO, and TOP1 provided by the FUSION software for five-fold cross-validation training, similar to the $3^{\prime}$aTWAS model.

In the association analysis stage, we select the model with the best prediction performance from the four models in the training stage. We extract the SNP-PDUI regression coefficients from this best-performing model to obtain the gene variation weight matrix $W={\mathrm{diag}}(W_{1},W_{2},\cdots ,W_{n})$. These coefficients weights are used directly in the kernel computation without any additional standardization, transformation, or adjustment applied to the weights themselves. We then calculate the kernel function $K_{w}$. Here, we use the weighted linear kernel function to represent $K_{w}$, as shown in the following formula:


(4) \begin{align*}& K_{w} = GWG^{T}\end{align*}

In the formulation, $W$ is the diagonal weight matrix extracted from the statistical model, $G$ is the genotype matrix, and $G_{{ij}}$ represents the information of the $j$th gene variant locus of the $i$th individual in the genotype matrix. Finally, we use the kernel function $K_{w}$ to perform the Q-score test, and the formula is as follows:


(5) \begin{align*}& Q = y^{T} K_{w} y\end{align*}

In the above formula, $y$ represents the trait vector. The Q-score test examines whether the assumption that the variance components explained by local genetic regions are uniformly zero is valid, where the Q-score follows a mixed chi-square distribution under the null hypothesis [16].

The ASTWAS model integrates the advantages of $3^{\prime}$aTWAS and kernel models. This method can pre-select gene variants from the perspective of APA in the $3^{\prime}$UTR of transcripts during the training phase, similar to $3^{\prime}$aTWAS, and can also use kernel models to aggregate linear and non-linear genetic effects of different variant sites during the association phase [17, 28].

### Data simulation procedure and power analysis

To comprehensively evaluate the statistical power of the ASTWAS and $3^{\prime}$aTWAS methods, this study conducted a large number of simulation experiments to systematically test and compare the performance of the two methods. This section first describes the data sources used in the simulation experiments and the corresponding preprocessing strategies. It then introduces the basic concepts of heritability and the theoretical basis for simulating phenotypic and transcriptomic data. The effect analysis and type I error adjustment section provides a detailed explanation of the design principles of type I error control experiments and how to evaluate model performance based on simulation results. Finally, it introduces how to apply the model to real-world datasets to identify potential susceptibility genes.

#### Genotype data and data preprocessing

The data used in the simulation experiment primarily come from the GTEx database and the 1KG database [34, 35]. We obtained genotype data, including both male and female individuals, from the 1000 Genomes (1KG) Project database, and then performed quality control on the obtained data. First, we performed missing data quality control, deleted SNPs with a missing rate >0.05, and also deleted individuals with a missing rate >0.05 in the sample. Then, we performed MAF quality control and removed SNPs with MAF <0.05 from the data. Similar processing was applied to the GTEx data, resulting in the retention of 670 samples and corresponding 4 500 300 genotypes from the GTEx dataset, and 2504 samples and corresponding 4 500 300 genotypes from the 1KG dataset.

In the simulation experiment, this paper trained the model on the processed GTEx dataset to obtain the weight information of each gene variant. The 1KG project dataset was used as the test dataset, and different models were used for association analysis on the test set to compare the statistical power of different models.

#### Heritability

The genetic central dogma states that the phenotypic traits of an individual undergo gene transcription to produce RNA, RNA translation to produce proteins, and proteins influence metabolic processes, ultimately affecting traits [36]. However, genes are also influenced by the environment during expression. Even individuals with the same gene sequence may express different traits in different environments. Therefore, gene expression is controlled by both genes and the environment [37], as expressed by the following formula:


(6) \begin{align*}& \mathrm{Phenotype} = \mathrm{Genotype} + \mathrm{Environment}\end{align*}

In the TWAS study, we use heritability to demonstrate the influence of genes on traits. Heritability is defined as the proportion of genetic variance to trait variance. The formula is as follows:


(7) \begin{align*}& h^{2} = \frac{\sigma_{g}^{2}}{\sigma_{p}^{2}}\end{align*}

In the formula, $\sigma _{g}^{2}$ is the genetic variance, $\sigma _{p}^{2}$ is the phenotypic variance, and $\sigma _{e}^{2}$ is the environmental variance. Traits are jointly influenced by genes and the environment, and the formula is expressed as:


(8) \begin{align*}& \sigma_{p}^{2} = \sigma_{g}^{2} + \sigma_{e}^{2}\end{align*}

The genetic component is applied in the simulation process to generate simulated phenotypic values corresponding to genes. In this study, the heritability is set to a preset value $h^2$ before simulation. Based on the given genetic variance $\sigma _{g}^{2}$, $\sigma _{p}^{2}$ can be obtained using Equation (7), and then $\sigma _{e}^{2}$ is calculated. A random sample is then drawn from the normal distribution $N(0,\sigma _{e}^{2})$ to determine the extent of the influence of non-genetic components such as noise on the phenotype. Finally, the sum of the genetic and non-genetic components is stored as the simulated phenotype.

#### Genetic assumptions and genetic structure

In order to comprehensively analyze, the statistical power of the $3^{\prime}$aTWAS and ASTWAS methods, this paper simulated the transcriptome and corresponding phenotype data for each sample based on the genotype data from the GTEx project and the 1000 Genomes Project, and then tested the association analysis effects of the two models on the simulated data. During the simulation process, this study primarily focused on two different genetic assumptions: causality and pleiotropy. Both genetic assumptions encompass multiple distinct genetic structures.

In the simulation process, this paper focuses on two genetic assumptions: causality and pleiotropy. Causality refers to the gene first affecting the length of the $3^{\prime}$UTR in the transcript, which then alters the phenotype of the individual. Pleiotropy refers to the gene simultaneously affecting the length of the $3^{\prime}$UTR in the transcript and the genetic structure of the individual’s traits. Under both genetic assumptions, the simulated phenotypes are associated with gene mutations. During the simulation process, we appropriately adjusted the simulated $3^{\prime}$UTR lengths and phenotypic values based on the value of the heritability to ensure that the simulated heritability effects were realistic, while also taking environmental factors into account.

Genetic structures can be generally classified into additive genetic structures and non-additive genetic structures. In additive genetic structures, this study uses the weighted sum of genetic effects to simulate transcriptomic $3^{\prime}$UTR length and phenotypic data. During the simulation process, we randomly selected $n$ SNPs from the genomic region $\sim \pm 150\mathrm{kb}$ around the gene coordinates. For each selected SNP, we randomly drew a value from a normal distribution $N(0,1)$ as the weight assigned to the SNP. Subsequently, we input the genetic effects of the selected SNPs and the preset heritability parameters into a specific formula to calculate the transcript data corresponding to each sample, thereby obtaining a simulated representation of the response of transcripts to potential genetic variation.

In the simulation experiments, this study selected $n=2,5,10$ cases to generate additive architecture simulation data. Nonadditive structures include four different genetic structures: Single, Epistatic, Heterogeneous, and Compensatory. The Single model is based on a single genotype, where a specific SNP variant locus influences transcriptomic data ($3^{\prime}$UTR length). Unlike the Single model, where only one SNP influences the simulation, the Epistatic, Heterogeneous, and Compensatory models randomly select two variants to influence the transcriptome and subsequent phenotypic data. Specifically, the Epistatic model requires that both SNPs carry variant alleles to alter the simulated data. However, in the Heterogeneous model, only one of the two SNPs needs to carry a mutated allele to alter the simulated data. In the Compensatory model, if only one of the two selected SNPs carries a mutated allele, the simulated data will be altered. However, if both SNPs carry mutated alleles, their effects will be mutually inhibitory and will not alter the simulated data. The genetic assumptions and genetic structure described above are presented in Fig. 1D.

#### Power calculation and adjustment to type I error

In the simulation experiments, this paper set up a total of seven genetic structures. For additive genetic structures, we set the number of variants with genetic effects in genes to $n=2,5,10$, respectively. In non-linear genetic structures, this paper set up four cases: Single, Epistatic, Heterogeneous, and Compensatory. In order to comprehensively compare the statistical effectiveness of the ASTWAS model and the $3^{\prime}$aTWAS model, and simulate the effects of gene inheritance on transcripts and phenotypes, this paper set different genetic strength parameters for causality and pleiotropy assumptions, as shown in Supplementary Table S1. In the causality assumption, we set five heritability parameters: 0.05, 0.1, 0.15, 0.2, and 0.25. In the pleiotropy assumption, we set six heritability parameters: 0.01, 0.02, 0.04, 0.06, 0.08, and 0.10. Therefore, we simulated and generated a total of 854 datasets, each containing 24 429 genes and 670 samples. During the simulation process, to facilitate the generation of transcriptomic and phenotypic data, we expanded the start site of each gene by 150 kb on either side, with causal variants randomly selected. We then tested the ability of the two models to successfully identify disease-causing genes in each dataset. In the simulated data, each gene is associated with its corresponding transcriptomic and phenotypic data. If the $P$-value of the association analysis results output by the model is lower than the predefined Bonferroni-corrected $P$-value threshold [38], we consider the model to have successfully identified the association of the current gene. The statistical power of the model’s association analysis is measured by the number of genes successfully identified by the model in a dataset divided by the total number of genes in the current dataset.

In statistics, a type I error is also known as a false positive, which occurs when a researcher incorrectly rejects a true hypothesis. In TWAS, a type I error refers to a model incorrectly identifying genes that are not associated with a phenotype as high-risk genes. Different TWAS models may exhibit inherent biases and high type I error rates due to differences in computational methods. Given a fixed threshold of 0.05, this study calculated the type I error rates of different methods to balance type I errors across all scenarios. In the simulation experiment, this study generated random transcriptomic and phenotypic data with no genetic components. Specifically, for each sample, a value was randomly generated from an $N(0,1)$ distribution to represent the transcriptomic and phenotypic data. The transcriptomic and phenotypic data of each simulated sample theoretically do not have any gene-related factors, which helps us confirm the zero distribution of each method. This paper compares the $3^{\prime}$aTWAS method with the ASTWAS method to detect the power of the two methods in identifying disease-causing genes in simulated data. We rank the significance values of each association analysis result from strong to weak and take the top 5$\%$ of the ranked values as the threshold for each method.

#### Real data testing and analysis

In addition to simulation experiments, this paper also compared the performance of the ASTWAS model and the $3^{\prime}$aTWAS model in two disease datasets: RA and T1D [39]. Each disease dataset included 2000 individuals primarily of European ancestry with genotypic data, along with an additional 3000 shared controls. The $3^{\prime}$TWAS project utilized the FUSION software to train models using reference datasets from ROS/MAP, PsychENCODE, and GTEx, resulting in 52 829 tissue-specific PDUI prediction models [40]. These 52 829 models encompassed 10 508 APA events across 7809 genes [28, 41]. In this study, the weight information from the PDUI prediction models was used for association analysis to validate the association between genes and diseases.

## Results

### Simulations

#### Type I errors and cutoffs

In whole-transcriptome association studies, while improving the model’s ability to identify susceptibility genes, it is also essential to avoid an excessively high rate of false positives. Therefore, controlling the type I error rate of the new model within a reasonable range is critical for the validity of the output results. To calculate the type I error rate of the new ASTWAS and $3^{\prime}$aTWAS models, this study set up a type I error simulation experiment. In the experiment, we randomly selected values from a standard normal distribution $N(0,1)$ as the transcriptome expression values or corresponding trait values for 24 760 genes in the sample. There was no association between genes and phenotypes in the simulated data. We tested the statistical efficacy of the ASTWAS and $3^{\prime}$aTWAS models on the simulated data. With a type I error rate defined as 5$\%$, the simulation results indicated that the type I error rate of the ASTWAS model was 0.0505, which was nearly identical to the target type I error rate. Similarly, the type I error rate of the $3^{\prime}$aTWAS model in the simulation experiments was 0.0514, also remaining near the 5$\%$ threshold. The above type I error simulation experiments indicate that the type I error rates of the ASTWAS and $3^{\prime}$aTWAS models are controlled within the expected range.

#### Pleiotropy hypothesis

**Figure 2:**
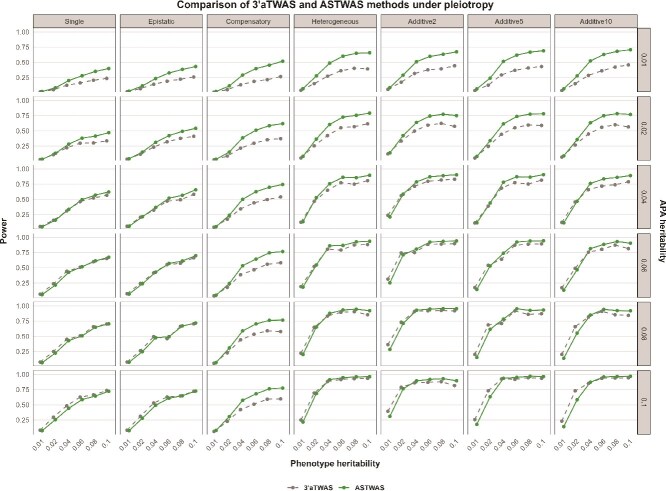
Statistical power of the ASTWAS and $3^{\prime}$aTWAS models under the pleiotropy assumption. The x-axis represents the phenotypic heritability, the left y-axis represents the statistical power of the model, and the right y-axis represents the APA heritability. Based on experience, we selected seven genetic structures, six phenotypic heritability values, and six APA heritability values, and simulated 252 data sets. The line graph provides a comprehensive assessment of the statistical power of $3^{\prime}$aTWAS and ASTWAS under different genetic conditions.

As can be seen from Fig. 2, under the pleiotropy hypothesis, the ASTWAS model suggests advantages in many scenarios. Under most genetic structures and heritability coefficients, the statistical power of the ASTWAS model is superior to that of the $3^{\prime}$aTWAS model. Under non-linear genetic structures, the ASTWAS model suggests notable advantages. The advantages of the ASTWAS model are more obvious when the genetic coefficients are low. As the genetic coefficients increase, the performance of the $3^{\prime}$aTWAS model also improves. Under epistatic and single genetic structures, when the genetic coefficients are highest, the ASTWAS model and the $3^{\prime}$aTWAS model show similar performance, and the $3^{\prime}$aTWAS model even slightly outperforms the ASTWAS model. However, overall, the ASTWAS model still has a significant advantage. Under the assumption of linear inheritance, the performance of the ASTWAS model is still better than that of the $3^{\prime}$aTWAS model. This advantage is more obvious when the heritability coefficient is low and the number of variants affecting the gene is small. Thanks to the advantage of the kernel method in capturing the nonlinear genetic effects of SNPs [16, 42], the ASTWAS model performed significantly better than the $3^{\prime}$aTWAS model in simulation experiments under the pleiotropy assumption.

#### Causality hypothesis

**Figure 3:**
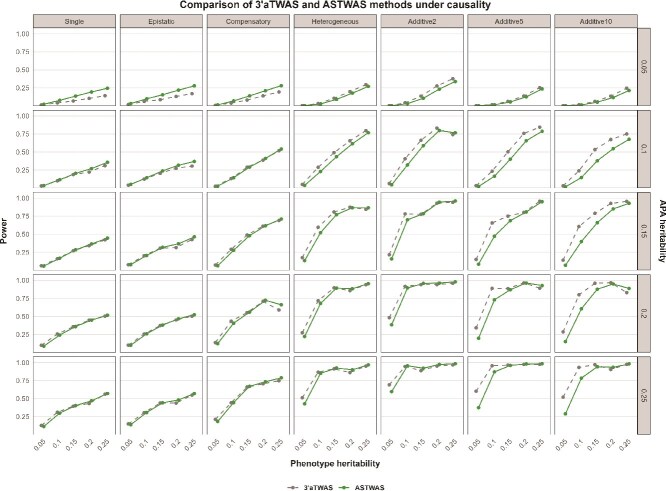
Statistical power of ASTWAS and $3^{\prime}$aTWAS models under causal genetic assumptions. The x-axis represents the phenotypic heritability, the left y-axis represents the statistical power of the model, and the right y-axis represents the APA heritability. Based on experience, this paper selected seven genetic structures, five phenotypic heritability values, and five APA heritability values to simulate 175 sets of data to comprehensively evaluate the statistical power of $3^{\prime}$aTWAS and ASTWAS under different genetic conditions.

As shown in Fig. 3, under the gene causality hypothesis, the ASTWAS model and the $3^{\prime}$aTWAS model have comparable association analysis capabilities, with each model demonstrating its own advantages in different genetic scenarios. Specifically, ASTWAS is superior to the $3^{\prime}$aTWAS model in most nonadditive scenarios, and this advantage decreases with increasing heritability. For example, under Single, Epistatic, and Compensatory genetic structures, the ASTWAS model shows better statistical power under various heritability coefficients. However, the situation is different under a heterogeneous genetic structure. When the genetic coefficient is low, the $3^{\prime}$aTWAS model shows a slight advantage, but this advantage disappears as the genetic coefficient increases. When the genetic coefficient is high, ASTWAS shows stronger statistical power. Under additive genetic structure, the $3^{\prime}$aTWAS model demonstrated excellent performance. This is because the $3^{\prime}$aTWAS model assumes a linear effect of genetic variation, using the weight coefficients calculated for each variation locus during association analysis to accumulate the effects of variation. Therefore, under the assumption of linear inheritance, the $3^{\prime}$aTWAS model showed significant advantages, but these advantages were more obvious when there were many genetic loci with effects. With the reduction of genetic effect loci in genes and the increase of the heritability coefficient, the advantages of the $3^{\prime}$aTWAS model decreased, and in some cases, the association effect of the ASTWAS model even surpassed that of the $3^{\prime}$aTWAS model.

#### Evaluating the impact of kernel selection on model power

We also evaluated the impact of kernel function selection on ASTWAS model performance, including the linear, linear weighted, quadratic, 2wayIX, IBS, and IBS weighted kernels. These evaluations were conducted under different genetic scenarios, including causality and pleiotropy, and covered both additive and nonadditive genetic architectures. The experimental results indicated that under both causality and pleiotropy scenarios, the linear weighted kernel consistently demonstrated the best or near-best power across all additive models (Heterogeneous, Additive2, Additive5, and Additive10). In all nonadditive models (Single, Epistatic, and Compensatory), the IBS weighted and quadratic kernels exhibited strong power. The 2wayIX kernel also demonstrated competitive power. The simulation results are presented in Supplementary Figs S3 and S4.

#### Impact of higher heritability on model power

To assess the robustness of our model across more heritability levels, we included five higher $h^{2}$ settings (0.10, 0.20, 0.40, 0.60, and 0.80) in addition to the original scenarios. These evaluations were conducted under both causality (Supplementary Fig. S1) and pleiotropy (Supplementary Fig. S2) assumptions, covering all seven genetic architectures. Under the pleiotropy assumption, ASTWAS consistently demonstrated higher power than $3^{\prime}$aTWAS in nonadditive architectures (Epistatic, Compensatory, and Heterogeneous), and this advantage became more pronounced as $h^{2}$ increased. Under the causality assumption, ASTWAS achieved slightly higher power in nonadditive settings, while both methods performed similarly under additive architectures.

#### Extending model evaluation to binary phenotypes

Since many target diseases are binary, we also ran simulations for case-control phenotypes. We implemented a liability-threshold framework to generate dichotomous traits and repeated all simulation scenarios. These evaluations were conducted under both causality (Supplementary Fig. S5) and pleiotropy (Supplementary Fig. S6) assumptions. The results for binary traits remained consistent with those for quantitative traits. Under pleiotropy, ASTWAS maintained a clear power advantage over $3^{\prime}$aTWAS, particularly in nonadditive architectures (Epistatic, Compensatory, and Heterogeneous). Under causality, both methods performed similarly in additive settings, while ASTWAS retained a slight edge in nonadditive settings, demonstrating the method’s robustness for case-control studies.

#### Impact of linkage disequilibrium structure on model power

To address the limitations of random SNP sampling, we implemented a linkage disequilibrium (LD)-aware simulation design. Instead of random selection, causal SNP sets were selected based on empirical intra-gene LD matrices, which we computed from the GTEx database’s gene-individual matrices using plink software. We set up two scenarios: a high-LD scenario (where causal SNP pairs had $r^{2}> 0.8$) and a low-LD scenario (where causal SNP pairs had $r^{2} < 0.2$), while preserving the realistic background LD structure of the region. These simulations were conducted under both causality and pleiotropy settings (Supplementary Figs S7 and S8). Results indicated that while both ASTWAS and $3^{\prime}$aTWAS showed higher power in the high-LD scenario, ASTWAS retained its clear advantage under pleiotropy. Furthermore, ASTWAS appeared less sensitive to the LD scenario under nonadditive causality scenarios, indicating greater robustness to varying local genetic architectures.

### Model performance on real datasets

#### Type 1 diabetes

**Figure 4:**
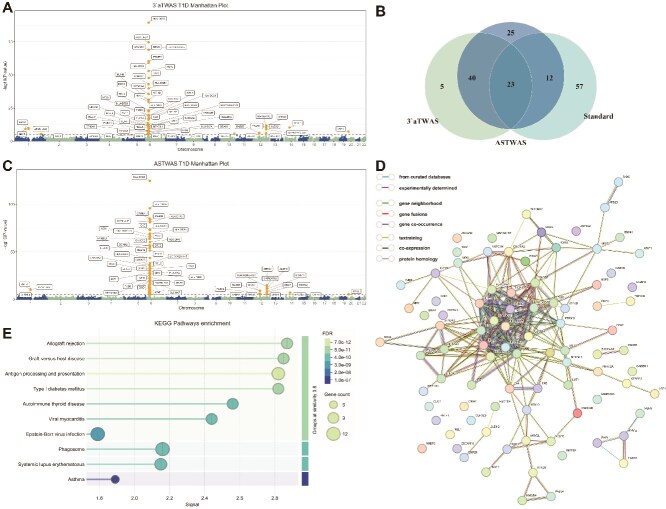
Analysis results of the ASTWAS model and $3^{\prime}$aTWAS model on T1D disease data. (A) Association results of the $3^{\prime}$aTWAS model on T1D disease. (B) Venn diagram of risk genes identified by the model. The Standard in the Venn diagram represents genes that have been identified as associated with T1D in the T1D data. Specifically, we collected genes associated with T1D from the DisGeNet database and genes associated with T1D from published literature, then intersected them with the 8000 genes we tested to obtain 92 genes, which were used as the Standard set. (C) Association results of the ASTWAS model on T1D disease. (D) Protein–protein interaction network of T1D susceptibility genes. Five interacting networks were identified, and a main network consisting of 62 TWAS susceptibility genes was determined, including T1D susceptibility genes such as *HLA-DRB5*, *PSMB9*, *HLA-B*, *HLA-A*, and *HLA-DPB1*, which have been validated by related studies. (E) KEGG pathway enrichment analysis identified pathways including Allograft rejection, Graft-versus-host disease, Antigen processing and presentation, and T1D mellitus.

T1D is a disease in which the patient is unable to produce insulin due to the destruction of his or her own pancreatic beta cells [43, 44]. Many people that have T1D are potentially at genetic risk, and close relatives of people with T1D have a higher risk to develop the disease, so exploring the genetic associations of T1D is important for the preventive diagnosis of the disease [45, 46]. T1D risk genes are shown in Supplementary Table S2. We applied the new models ASTWAS and $3^{\prime}$aTWAS to the Wellcome Trust Case Control Consortium (WTCCC) T1D dataset [39]. The two models identified a total of 103 susceptibility genes associated with T1D ($P<6.15\times 10^{-6}$). The association analysis results of the ASTWAS model are shown in Fig. 4C, which identified 100 susceptibility genes. Figure 4A shows the susceptibility genes for T1D identified by the $3^{\prime}$aTWAS model, which identified 66 susceptibility genes, 63 of which were already included in the ASTWAS model results.

The susceptibility genes discovered by the ASTWAS model were mainly located within seven regions, 1p13.2, 6p21.32, 6p21.33, 6p22.1, 6p22.2, 12q13.3, 12q24.12, and *CNIH1* ($P=5.71\times 10^{-11}$) at 14q22.2 and *SNX5* ($P=6.13\times 10^{-8}$) at 20p11.23 were also identified. The $3^{\prime}$aTWAS model identified risk loci mainly within the regions 1p13.2, 6p21.32, 6p21.33, 6p22.1, 6p22.2, 12q13.2, 12q13.3, 12q24.12, 12q24.13, and also identified the *GBP5* ($P=4.71\times 10^{-10}$) located in the 1p22.2 region, *NSL1* ($P=5.33\times 10^{-6}$) located in the 1q32.3 region, *INPP5F* ($P=2.50\times 10^{-6}$) in the 10q26.11 region, *CNIH1* ($P=5.71\times 10^{-11}$) in the 14q22.2 region, and the *SNX5* ($P=6.13\times 10^{-8}$) in the 20p11.23 region.

In the 37 susceptibility genes identified exclusively by the ASTWAS model, 29 genes are located near the MHC region, 3 genes are located in the 12q13.3 region, 4 genes are located in the 12q24.11, 12q24.12, and 12q24.13 regions, and *OLFML3* gene is located in the 1p13.2 region. Among the 37 susceptibility genes, 12 were previously identified as T1D susceptibility genes in prior studies, while 25 genes were newly identified [8, 47–56]. Among the newly identified genes, 20 were located within 1 Mb of genes associated with T1D, and 5 genes (*OLFML3*, *VPS29*, *BRAP*, *HECTD4*, and *PTPN11*) lacked evidence supporting their association with T1D. The *OLFML3* gene is a protein-coding gene associated with diseases such as Alzheimer’s disease, frontotemporal dementia, and amyotrophic lateral sclerosis [57, 58]. *VPS29* is a protein-coding gene associated with diseases such as Ritscher–Schinzel syndrome and late-onset Parkinson’s disease [59, 60]. Its related pathways include serine and glycine biosynthesis and WNT signaling. *BRAP* is a protein-coding gene, and diseases associated with *BRAP* include myocardial infarction, cardiovascular diseases, and metabolic syndrome [61–63]. The *HECTD4* gene is a protein-coding gene. Carriers of the *HECTD4* SNP alleles (rs77768175, rs2074356, and rs11066280) have significantly lower fasting blood glucose levels than non-carriers. Studies have found that *HECTD4* polymorphism has a protective effect on the risk of diabetes in drinkers [64]. *PTPN11* is a protein-coding gene. Diseases associated with *PTPN11* include Noonan syndrome 1 and juvenile myelomonocytic leukemia. Related studies indicate that linoleic acid is a multi-target inhibitor of *PTPN1*, *PTPN9*, and *PTPN11*, potentially exerting an anti-diabetic effect to prevent type 2 diabetes [65]. It remains unclear whether it can influence the development of T1D.

It is worth noting that the susceptibility gene *GABBR1* identified exclusively by the ASTWAS model has not yet been associated with T1D in any TWAS studies. The *GABBR1* gene encodes the $\gamma $-aminobutyric acid (*GABA*) receptor. *GABA* is the primary inhibitory neurotransmitter in the mammalian central nervous system [53]. Recent advances have highlighted the multifaceted role of *GABA* in regulating pancreatic endocrine function, enhancing $\beta $-cell proliferation, and protecting islet integrity through immunomodulatory effects. Preclinical studies and early clinical trials suggest that *GABA*, whether administered alone or in combination with other therapeutic agents, may restore $\beta $-cell function and improve glycemic control. Additionally, *GABA* is involved in alleviating T1D-related complications, such as diabetic nephropathy, neuropathy, retinopathy, and cognitive decline [66]. Therefore, the *GABBR1* gene may influence the development of T1D, and this influence is mediated through *GABA* transmission.

#### Protein–protein interaction network and enrichment analysis with susceptibility genes of type 1 diabetes

In this study, we performed protein–protein interaction network and enrichment analysis on the T1D susceptibility genes output by the ASTWAS model. As shown in Fig. 4D, we identified a total of five networks, one of which is a main network consisting of 62 T1D susceptibility genes, mainly connected by T1D susceptibility genes such as *HLA-DRB5*, *HLA-B*, *HLA-A*, and *HLA-DPB1*, which have been validated by relevant studies. The remaining four interconnected networks are as follows: one network comprising four genes, one network comprising three genes, and two networks each containing only two interconnected genes.

We performed a KEGG pathway enrichment analysis on the T1D susceptibility genes output by the ASTWAS model. The KEGG enrichment analysis results not only directly identified T1D but also pointed to its core autoimmune disease pathways. The KEGG enrichment results are shown in Fig. 4E. Among the enriched pathways, immune-related pathways such as Allograft rejection, Graft-versus-host disease, Antigen processing and presentation, T1D mellitus, and Autoimmune thyroid disease (AITD showed high enrichment significance (FDR far <0.05). In particular, the T1D pathway (${\mathrm{FDR}} = 3.60\times 10^{-11}$) is directly related to the target disease of the study, indicating that our model can effectively identify gene networks directly related to the disease and effectively identify susceptibility genes closely related to the pathogenesis. The most significant pathway (the lowest FDR value) in the enrichment results was antigen processing and presentation. This is closely related to the pathogenesis of T1D. Under pathological conditions, pancreatic $\beta $ cells possess antigen presentation functions, capable of processing and presenting self-antigens, activating self-reactive T cells, and actively participating in the initiation of autoimmune responses, highlighting the role of this pathway in T1D pathogenesis [67]. Allograft rejection and graft-versus-host disease pathways are associated with immune responses involving T cells, similar to T1D mellitus. The AITD pathway is significantly enriched, consistent with clinical observations that T1D and AITD share genetic susceptibility genes. The two diseases often co-occur in individuals and families, share the same *HLA* genetic background, and have common susceptibility loci for multiple immune regulatory genes, reflecting the genetic association between autoimmune diseases [68]. The significance of the systemic lupus erythematosus pathway is consistent with the conclusion of a Mendelian randomization study confirming a causal relationship between T1D and SLE [69, 70].Viruses are one of the environmental factors contributing to the onset of T1D [70, 71]. KEGG enrichment analysis showed that the Epstein-Barr virus infection pathway was significantly enriched. This may be because the *EBV* protein *BOLF1* shares sequence homology with the *HLA-DQw8* beta chain, and the repeated pentapeptide sequence in the *EBV-BERF4* epitope may induce cross-reactive immune responses, leading to the destruction of self-pancreatic $\beta $ cells [72, 73].

Enrichment analysis of T1D susceptibility genes identified by the ASTWAS model. The ASTWAS model not only accurately identified T1D-specific pathways but also revealed the underlying immune mechanisms, other related autoimmune diseases, and potential environmental factors. This fully demonstrates the powerful association analysis ability of the ASTWAS model and its reliability in identifying the true susceptibility genes for complex diseases.

#### Rheumatoid arthritis

Rheumatoid arthritis (RA) is a systemic autoimmune disease characterized by inflammatory arthritis and bursal involvement [74, 75]. It is often caused by an interaction between genetic and environmental factors (including tobacco), primarily affecting synovial joints [76].

We also applied the model to the RA dataset from the WTCC. The RA susceptibility genes identified by the ASTWAS model and the $3^{\prime}$aTWAS model are shown in Supplementary Table S3. The ASTWAS model identified 56 high-risk genes, while the $3^{\prime}$aTWAS model identified 44 high-risk genes, of which 41 were already included in the ASTWAS model results.

In the 15 genes identified exclusively by the ASTWAS model, we found that 6 genes have been confirmed to be associated with RA [77–83]. The *SLC39A7* gene was identified as a candidate gene associated with rheumatic diseases in related GWAS studies, but it is unclear whether it is involved in the genetic process of RA [82]. The *TCF19* gene ($P=2.57\times 10^{-7}$) has been significantly associated with various autoimmune diseases and human cancers (including cervical cancer and autoimmune thyroiditis), but its association with RA remains unclear. It may establish an association with RA by influencing the immune system. The *DDR1* gene plays a key role in numerous signaling pathways involved in bone formation, regeneration, and cartilage-related diseases in humans [84]. *DDR1* expression disorders can delay bone growth and bone regeneration, but its relationship with RA remains unclear. The *RGL2* gene is involved in the “Immune response Antigen presentation by MHC class II” pathway, and a TWAS study has suggested it as a risk gene for RA. However, the association between this gene and RA disease remains uncertain [85]. The remaining 5 genes (*NUP98*, *DXO*, *PSMB8-AS1*, *NFKBIL1*, and *PRRC2A*) have no known literature supporting a potential association with RA.

#### Protein–protein interaction network and enrichment analysis with susceptibility genes of rheumatoid arthritis

**Figure 5:**
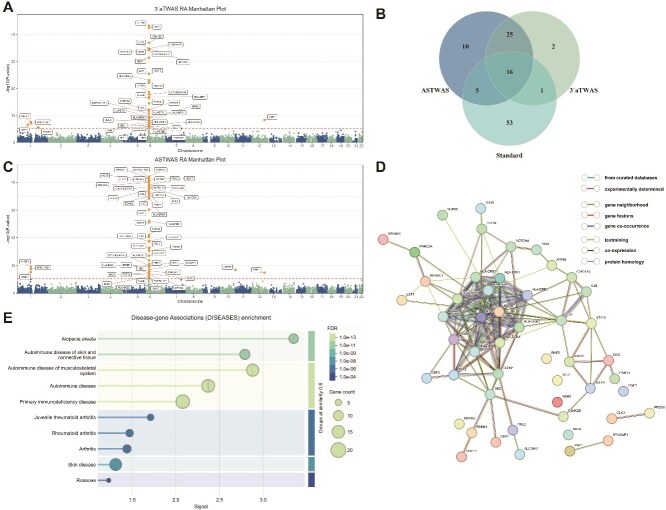
Analysis results of the ASTWAS model and $3^{\prime}$aTWAS model on RA disease data. (A) Association results of the $3^{\prime}$aTWAS model on RA disease. (B) Venn diagram of risk genes identified by the model. Overlap of association results of the two models on RA disease. (C) Association results of the ASTWAS model on RA disease. (D) Protein–protein interaction network of RA susceptibility genes. A main network consisting of 40 RA susceptibility genes was identified, including the well-known RA susceptibility genes *HLA-DRB1*, *HLA-C*, *HLA-B*, and *HLA-DQB1*. (E) Gene–disease association enrichment analysis identified diseases such as alopecia areata, musculoskeletal system autoimmune diseases, skin and connective tissue immune diseases, primary immunodeficiency disorders, juvenile RA, and RA. A multi-panel figure labeled (A) to (E) showing analysis results for T1D. Panels (A) and (C) are Manhattan plots displaying significant genetic signals on chromosome 6. Panel (B) is a Venn diagram showing the overlap of significant genes identified by three different methods. Panel (D) is a gene interaction network graph. Panel (E) is a dot plot showing KEGG pathway enrichment with terms such as T1D mellitus labeled on the y-axis.

To further investigate the potential biological mechanisms underlying RA disease susceptibility genes, this study utilized STRING [86] to generate a protein–protein interaction network for the TWAS-identified susceptibility genes and performed enrichment analysis. As shown in Fig. 5D, we identified a main network consisting of 40 RA susceptibility genes, including well-known RA susceptibility genes *HLA-DRB1*, *HLA-C*, *HLA-B*, and *HLA-DQB1* [87–89]. Two additional networks were identified, each comprising two susceptibility genes. In the *CLIC1*-*PRDX6* network, *CLIC1* activates the inflammasome in RA [90].

Disease–gene association enrichment analysis was performed on the susceptibility genes identified by the ASTWAS model in the RA dataset, with the results shown in Fig. 5E. The enrichment analysis results indicated that alopecia areata exhibited the highest enrichment signal (${\mathrm{FDR}}=1.07\times 10^{-11}$), followed by other enrichment results such as musculoskeletal system autoimmune diseases (${\mathrm{FDR}}=3.22\times 10^{-13}$), skin and connective tissue autoimmune diseases (${\mathrm{FDR}}=7.19\times 10^{-11}$), autoimmune diseases (${\mathrm{FDR}}=1.79\times 10^{-12}$), primary immunodeficiency diseases (${\mathrm{FDR}}=1.69\times 10^{-12}$), juvenile RA (${\mathrm{FDR}}=7.10\times 10^{-6}$), RA (${\mathrm{FDR}}=1.50\times 10^{-5}$), arthritis (${\mathrm{FDR}}=8.53\times 10^{-6}$), and skin diseases (${\mathrm{FDR}}=1.78\times 10^{-7}$).rosacea (${\mathrm{FDR}}=3.4\times 10^{-4}$). The significant enrichment of alopecia areata, rosacea, skin diseases, and skin and connective tissue autoimmune diseases reflects the comorbidity between RA and skin system diseases. This finding is consistent with clinical observations that RA patients often have multiple skin autoimmune diseases, suggesting that these diseases may share similar genetic susceptibility mechanisms [91, 92]. The significant enrichment of diseases ranging from broad autoimmune diseases to musculoskeletal immune diseases and primary immunodeficiency diseases reflects the systemic autoimmune nature of RA [93]. The significant enrichment of juvenile RA, RA, and arthritis is related to the target traits of the model study, indicating that our model can effectively identify susceptibility genes for target traits. In particular, the enrichment of RA provides direct evidence for the accuracy of the model results. Gene–disease association enrichment experiments provide support for the accuracy and biological validity of the RA susceptibility genes identified by the ASTWAS model, and support the association analysis capabilities of the ASTWAS model from multiple dimensions.

## Conclusion and discussion

In this study, we developed ASTWAS, a framework designed to address potential limitations of linear aggregation in prior APA-TWAS models. By integrating multiple APA usage prediction models (BLUP, Elastic Net, LASSO, and TOP1) with a weighted sequence kernel association test, our approach is intended to capture both additive and nonadditive genetic contributions, which may be overlooked by linear models.

We compared the statistical power of ASTWAS and $3^{\prime}$aTWAS under various simulated genetic architectures and heritability levels. The simulation results suggest that ASTWAS may offer performance advantages over linear APA-TWAS across diverse genetic architectures, particularly under scenarios of pleiotropy and lower heritability. When applied to WTCCC T1D and RA cohorts, ASTWAS not only recapitulated known risk loci but also identified novel candidate genes. Subsequent network and pathway analyses of these candidates highlighted their relevance to key immune mechanisms, underscoring the potential biological plausibility of our approach.

Despite these promising results, ASTWAS possesses several limitations. First, a significant number of identified susceptibility genes for T1D and RA reside within the highly complex MHC region. Consequently, some observed associations may reflect complex LD rather than independent causal effects. Conditional analyses will be necessary in future studies to disentangle these signals and establish which genes represent independent risk loci. Second, the current ASTWAS implementation requires individual-level genotype and APA data for model training. This presents a practical barrier to its application compared with methods leveraging summary statistics, which can benefit from much larger reference panels. Finally, rare variants may materially influence gene expression and APA [94, 95], the present implementation considers only common SNPs; incorporating rare-variant information is therefore an important limitation and a priority for future extensions.

Recent work indicates that polygenic scores (PGS) can aggregate small SNP effects and improve cross-cohort robustness, suggesting a natural path to improve ASTWAS [96, 97]. OTTERS, for example, has shown that adapting PRS methods to derive eQTL weights from summary-level reference data can enhance TWAS power and cross-cohort robustness [14]. A future ASTWAS framework could adopt a similar strategy, using summary-level $3^{\prime}$aQTL data to train robust, PGS-style weights for APA usage. This approach could overcome the need for individual-level data and improve statistical power by using large-scale public reference datasets, which broaden the application of ASTWAS for complex trait discovery. In the future, we will also incorporate rare variants through burden or SKAT-type aggregation at gene or regulatory units, leverage richer functional annotations such as conservation and pathogenicity scores, regulatory and splicing features, APA motifs, and RBP or miRNA binding sites to guide variant weights and kernel design, and introduce PGS priors or auxiliary features to improve cross-cohort robustness and gene-level signal recovery.

**Key Points**

- We propose ASTWAS, a nonlinear APA transcriptome-wide association study method that uses a kernel-based framework to integrate APA quantitative trait loci, capturing both additive and nonadditive genetic effects.
- Thorough testing of ASTWAS against $3^{\prime}$aTWAS across two genetic assumptions and four architectures with varying heritability revealed that ASTWAS consistently achieves greater statistical power, particularly when heritability is low.
- When applied to type 1 diabetes and rheumatoid arthritis cohorts, ASTWAS not only discovered more literature-supported risk genes than $3^{\prime}$aTWAS but also identified novel candidate susceptibility genes.
- We performed protein–protein interaction (PPI) network and enrichment analyses on the susceptibility genes identified by the ASTWAS model. These analyses not only validated the accuracy and biological relevance of the results but also provided insights into the underlying pathological mechanisms of the diseases.

## Supplementary Material

20251222_supplementary_ASTWAS-ALT-TXT_bbaf725

## Data Availability

The ASTWAS model in this paper is implemented entirely using Python code. The program does not require any other software and can independently complete the association analysis calculation process. The source code for the ASTWAS model can be obtained from https://github.com/wl-Simplecss/ASTWAS.
