# Supplementary material for: ASTWAS: modeling alternative polyadenylation and SNP effects in kernel-driven TWAS reveal novel genetic associations for complex traits
Source: Brief Bioinform. 2026 Jan 19;27(1):bbaf725. doi: 10.1093/bib/bbaf725 (PMC12814985; doi:10.1093/bib/bbaf725)
Supplement: 20251222_supplementary_ASTWAS-ALT-TXT_bbaf725 [file 20251222_supplementary_astwas-alt-txt_bbaf725.docx]

**ASTWAS: Modeling alternative polyadenylation and SNP Effects in Kernel-Driven TWAS reveals novel genetic associations for complex traits**

Yan Wang^1^, Lei Wang^1,2^, Nan Sheng^1^, Jie Hong^1^, Yunzhi Liu^1^, Pengze Wu^3^, XinFei Wang^1^, Shuyan Zhang^4,5^, Chen Cao^3,*^

*1 Key laboratory of Symbol Computation and Knowledge Engineering of Ministry of Education, College of Computer Science and Technology, Jilin University, 130012, Changchun, China,*

*2 Shenzhen Loop Area Institute, 518045, Shenzhen, China,*

*3 School of Biomedical Engineering and Informatics, Nanjing Medical University, 211166, Nanjing, China,*

*4 National Key Laboratory of Intelligent Tracking and Forecasting for Infectious Diseases, Beijing Ditan Hospital, Capital Medical University, 100015, Beijing, China*

*5 Beijing Institute of Infectious Diseases, 100015, Beijing, China*

∗ Corresponding author.

Email: caochen@njmu.edu.cn (Cao C)

**Supplementary Table 1 (ST1).** Selection of simulation experiment parameters.

| Genetic Assumption | Genetic Architecture | $\boldsymbol{h}_{\mathbf{transcriptome}}^{\mathbf{2}}$ | $\boldsymbol{h}_{\mathbf{phenotype}}^{\mathbf{2}}$ |
| --- | --- | --- | --- |
| Pleiotropy | Single, Epistatic, Heterogeneous, Compensatory, Additive2, Additive5, Additive10 | 0.01, 0.02, 0.04, 0.06, 0.08, 0.10 | 0.01, 0.02, 0.04, 0.06, 0.08, 0.10 |
| Causality | Single, Epistatic, Heterogeneous, Compensatory, Additive2, Additive5, Additive10 | 0.05, 0.10, 0.15, 0.20, 0.25 | 0.05, 0.10, 0.15, 0.20, 0.25 |

**Supplementary Table 2(ST2).** Susceptibility genes identified by the ASTWAS and 3`aTWAS models in the T1D dataset. (A question mark after the literature ID indicates that there is a study suggesting an association risk between the gene and another gene, but the association between the two has not yet been proven.)

| Gene | Chr | Start | End | ASTWAS_P | 3`aTWAS_P | Reference | MalaCards |
| --- | --- | --- | --- | --- | --- | --- | --- |
| ***HLA-DRB6*** | chr6 | 32553046 | 32558186 | 2.59E-125 | 7.63E-60 | PMID:38887913 |  |
| ***HLA-DRB5*** | chr6 | 32517353 | 32530287 | 1.30E-96 | 3.00E-90 | PMID:37528512 |  |
| ***GPANK1*** | chr6 | 2917047 | 2922107 | 6.88E-86 | 1.06E-69 | PMID:33179336? |  |
| ***PSMB9*** | chr6 | 32844136 | 32860734 | 1.13E-84 | 4.88E-61 | PMID:37528512 | TRUE |
| ***CYP21A1P*** | chr6 | 3306878 | 3309588 | 1.19E-82 | 6.18E-06 |  |  |
| ***HLA-DPA1*** | chr6 | 4363634 | 4379828 | 6.31E-82 | 8.91E-24 |  |  |
| ***C4B*** | chr6 | 3324061 | 3324087 | 2.14E-74 | 2.53E-17 | PMID:22151770 |  |
| ***HLA-DQB1*** | chr6 | 4076917 | 4086211 | 8.42E-74 | 1.41E-40 | PMID:34529725 | TRUE |
| ***HLA-DRB1*** | chr6 | 32577902 | 32589848 | 5.13E-70 | 2.24E-75 | PMID:34153873 | TRUE |
| ***LOC100507547*** | chr6 | 32152802 | 32154365 | 1.38E-68 | 1.06E-69 |  |  |
| ***NEU1*** | chr6 | 31857063 | 31862905 | 2.25E-66 | 1.06E-69 | PMID:23520133? |  |
| ***PBX2*** | chr6 | 3489722 | 3495164 | 2.82E-66 | 1.07E-53 | PMID:33179336 |  |
| ***NFKBIL1*** | chr6 | 31546870 | 31558829 | 9.05E-65 | 2.04E-10 |  |  |
| ***SNHG32*** | chr6 | 31822094 | 31839766 | 1.35E-64 | 1.85E-11 |  |  |
| ***HLA-DMA*** | chr6 | 4142920 | 4165041 | 4.59E-61 | 4.77E-09 | PMID:10519369 | TRUE |
| ***DDAH2*** | chr6 | 3032085 | 3035664 | 1.21E-57 | 4.13E-08 |  |  |
| ***HLA-C*** | chr6 | 2577801 | 2581172 | 1.36E-57 | 2.00E-30 | PMID:31152121 |  |
| ***BRD2*** | chr6 | 32968594 | 32981505 | 9.59E-57 | 5.55E-24 |  |  |
| ***NELFE*** | chr6 | 3294123 | 3301128 | 4.40E-55 | 6.46E-17 | PMID:37528512 |  |
| ***STK19*** | chr6 | 31971091 | 31982821 | 2.56E-52 | 4.97E-06 |  |  |
| ***HLA-DQA1*** | chr6 | 3836385 | 3842664 | 1.77E-44 | 1.21E-19 | PMID:26854762 | TRUE |
| ***LOC100294145*** | chr6 | 32894144 | 32903758 | 6.91E-39 | 2.03E-13 |  |  |
| ***CSNK2B*** | chr6 | 2970283 | 2977329 | 4.21E-37 | 5.51E-12 |  |  |
| ***CYP21A2*** | chr6 | 3380335 | 3383703 | 1.39E-36 | 2.34E-36 | PMID:34529725 | TRUE |
| ***RGL2*** | chr6 | 4727590 | 4735260 | 2.10E-35 | 7.09E-20 |  |  |
| ***DDR1*** | chr6 | 2346729 | 2355691 | 6.43E-34 | 8.60E-32 | PMID:32398868? |  |
| ***TAP2*** | chr6 | 32821833 | 32838739 | 5.13E-33 | 4.15E-50 | PMID:17192492 | TRUE |
| ***MICA*** | chr6 | 31382711 | 31400793 | 1.67E-31 | 5.79E-20 | PMID:16698430 | TRUE |
| ***HLA-B*** | chr6 | 31353872 | 31367067 | 2.67E-31 | 6.64E-32 | PMID:19143813 | TRUE |
| ***HLA-DRA*** | chr6 | 3662891 | 3668101 | 5.85E-31 | 2.60E-10 | PMID:34220961 |  |
| ***NRM*** | chr6 | 1998300 | 2001673 | 5.90E-31 | 6.53E-08 |  |  |
| ***LST1*** | chr6 | 2891165 | 2893950 | 6.05E-29 | 9.48E-12 | PMID:38539228 |  |
| ***HLA-DPB1*** | chr6 | 33075936 | 33089696 | 8.48E-28 | 1.67E-20 | PMID:20424227 | TRUE |
| ***TCF19*** | chr6 | 31158331 | 31167159 | 2.06E-26 | 1.00E-17 | PMID:21076979 |  |
| ***RNF5*** | chr6 | 3483335 | 3485774 | 3.88E-24 | 3.14E-03 |  |  |
| ***TUBB*** | chr6 | 2030452 | 2035677 | 2.46E-23 | 3.25E-23 |  |  |
| ***PSMB8-AS1*** | chr6 | 4263439 | 4265848 | 3.06E-22 | 4.20E-01 |  |  |
| ***MRPS18B*** | chr6 | 1927938 | 1936624 | 5.93E-21 | NA |  |  |
| ***SLC39A7*** | chr6 | 33200305 | 33204439 | 1.67E-20 | 9.79E-03 |  |  |
| ***DXO*** | chr6 | 3311846 | 3314328 | 3.57E-20 | 9.55E-08 |  |  |
| ***ATAT1*** | chr6 | 30626842 | 30646823 | 6.15E-20 | 1.66E-06 |  |  |
| ***COL11A2*** | chr6 | 33162681 | 33192499 | 3.29E-18 | 9.46E-24 |  |  |
| ***ATF6B*** | chr6 | 3457284 | 3470273 | 5.50E-17 | 5.09E-32 |  |  |
| ***C4A*** | chr6 | 3374766 | 3378295 | 1.83E-16 | 2.06E-04 | PMID:22151770 | TRUE |
| ***SAPCD1-AS1*** | chr6 | 3106199 | 3107477 | 4.34E-16 | 5.14E-05 |  |  |
| ***ERP29*** | chr12 | 112013348 | 112023449 | 9.82E-16 | 1.63E-12 |  |  |
| ***ABT1*** | chr6 | 26596953 | 26600739 | 1.12E-15 | 2.59E-14 |  |  |
| ***MAPKAPK5*** | chr12 | 111842013 | 111902222 | 4.99E-15 | 1.29E-11 |  |  |
| ***H2AC6*** | chr6 | 26124145 | 26139116 | 1.03E-14 | 7.81E-01 |  |  |
| ***MAPKAPK5-AS1*** | chr12 | 111839758 | 111842902 | 2.02E-14 | 1.21E-09 |  |  |
| ***HMGN4*** | chr6 | 26538366 | 26546933 | 2.84E-14 | 4.64E-13 | 10.4161/epi.26407? |  |
| ***BTN3A1*** | chr6 | 26402237 | 26415208 | 6.88E-14 | 4.85E-08 | PMID:39417845 |  |
| ***SKIV2L*** | chr6 | 31959116 | 31969818 | 1.85E-13 | 5.27E-02 |  |  |
| ***TAPBP*** | chr6 | 33299694 | 33314284 | 3.47E-13 | 2.21E-04 | PMID:36289205? |  |
| ***BTN3A3*** | chr6 | 26440472 | 26453415 | 5.08E-13 | 3.48E-06 |  |  |
| ***ALDH2*** | chr12 | 111766887 | 111817532 | 2.16E-12 | 3.02E-11 | PMID:34725563 |  |
| ***TRIM38*** | chr6 | 25962802 | 25991231 | 2.24E-12 | 2.22E-04 |  |  |
| ***PTPN11*** | chr12 | 82580117 | 82581384 | 6.21E-12 | 8.09E-04 | PMID:30245508 |  |
| ***PRRC2A*** | chr6 | 31620715 | 31637771 | 1.15E-11 | 3.14E-03 | PMID:35784577 |  |
| ***PHTF1*** | chr1 | 113696831 | 113759489 | 1.97E-11 | 1.56E-10 | PMID:34234497 |  |
| ***HFE*** | chr6 | 26087226 | 26098343 | 3.08E-11 | 5.04E-06 |  |  |
| ***PHF1*** | chr6 | 4604785 | 4605630 | 3.22E-11 | 3.13E-06 |  |  |
| ***ZNF322*** | chr6 | 26634383 | 26659752 | 3.65E-11 | 1.03E-06 |  |  |
| ***ZSCAN16-AS1*** | chr6 | 28008599 | 28137319 | 7.46E-11 | 2.34E-05 |  |  |
| ***AP4B1-AS1*** | chr1 | 113812379 | 113901237 | 9.77E-11 | 6.70E-10 | PMID:34521982 |  |
| ***ZSCAN16*** | chr6 | 28107689 | 28130082 | 1.09E-10 | 2.74E-04 | PMID:26258848? |  |
| ***NOTCH4*** | chr6 | 3418042 | 3447269 | 1.13E-10 | 3.01E-02 |  |  |
| ***MSH5*** | chr6 | 3217300 | 3242109 | 1.78E-10 | 6.39E-02 |  |  |
| ***PGBD1*** | chr6 | 28281572 | 28302549 | 4.73E-10 | 1.15E-08 |  |  |
| ***RNF41*** | chr12 | 56202179 | 56221933 | 4.89E-10 | 1.65E-06 |  |  |
| ***CLIC1*** | chr6 | 31730581 | 31739763 | 5.20E-10 | 3.01E-02 | PMID:38327933? |  |
| ***RAB5B*** | chr12 | 55973913 | 55996683 | 5.38E-10 | 5.89E-07 | 10.1007/978-3-030-02116-0 |  |
| ***HLA-A*** | chr6 | 29941260 | 29949572 | 6.46E-10 | 1.77E-11 | PMID:19143813 | TRUE |
| ***HLA-J*** | chr6 | 1304413 | 1307346 | 8.32E-10 | 1.76E-12 |  |  |
| ***BTN2A1*** | chr6 | 26457904 | 26476621 | 1.41E-09 | 1.67E-08 |  |  |
| ***BRAP*** | chr12 | 111642146 | 111685956 | 1.88E-09 | 1.82E-03 |  |  |
| ***BAK1*** | chr6 | 33572547 | 33580293 | 3.38E-09 | 4.57E-01 |  |  |
| ***HLA-H*** | chr6 | 29887565 | 29924658 | 3.92E-09 | 7.27E-12 |  |  |
| ***ZSCAN9*** | chr6 | 28224886 | 28233487 | 5.54E-09 | 4.63E-02 |  |  |
| ***BTN2A2*** | chr6 | 26383096 | 26394874 | 5.83E-09 | 8.88E-03 | PMID:39417845 |  |
| ***ZKSCAN4*** | chr6 | 28241697 | 28252269 | 7.20E-09 | 3.10E-02 | PMID:26258848? |  |
| ***HLA-V*** | chr6 | 29792234 | 29793136 | 7.47E-09 | 7.66E-11 |  |  |
| ***PHETA1*** | chr12 | 111360651 | 111369121 | 1.76E-08 | 1.19E-06 |  |  |
| ***H2BC4*** | chr6 | 26086317 | 26123926 | 2.02E-08 | 4.21E-02 |  |  |
| ***HLA-DQB2*** | chr6 | 3950399 | 3957838 | 2.48E-08 | 2.98E-18 |  |  |
| ***CNIH1*** | chr14 | 54423561 | 54441391 | 5.33E-08 | 5.71E-11 |  |  |
| ***PRRT1*** | chr6 | 32148359 | 32153083 | 1.21E-07 | 3.08E-05 |  |  |
| ***VPS52*** | chr6 | 4444672 | 4466464 | 3.34E-07 | 1.54E-01 |  |  |
| ***PA2G4*** | chr12 | 56104537 | 56113910 | 3.90E-07 | 2.62E-04 | PMID:21850031 |  |
| ***NABP2*** | chr12 | 56222015 | 56229854 | 4.25E-07 | 1.43E-03 |  |  |
| ***HLA-F-AS1*** | chr6 | 29707518 | 29828496 | 9.29E-07 | 4.00E-02 |  |  |
| ***OLFML3*** | chr1 | 113979391 | 114035572 | 1.03E-06 | 5.96E-02 |  |  |
| ***HECTD4*** | chr12 | 112160188 | 112382439 | 1.23E-06 | 6.92E-04 |  |  |
| ***RPS26*** | chr12 | 56041351 | 56044697 | 1.32E-06 | 2.26E-04 | PMID:38041572 |  |
| ***VPS29*** | chr12 | 110491083 | 110502111 | 1.61E-06 | 1.92E-05 |  |  |
| ***PPP1R11*** | chr6 | 1321869 | 1325493 | 1.67E-06 | 1.67E-05 | PMID:30882958? |  |
| ***MOG*** | chr6 | 922481 | 937873 | 2.75E-06 | 6.62E-06 |  |  |
| ***GABBR1*** | chr6 | 29555629 | 29633976 | 2.75E-06 | 2.37E-05 | PMID:37969011 |  |
| ***SNX5*** | chr20 | 17941597 | 17968980 | 2.79E-06 | 6.13E-08 |  |  |
| ***RXRB*** | chr6 | 4387991 | 4395092 | 2.99E-06 | 1.32E-05 |  |  |
| ***GBP5*** | chr1 | 89256189 | 89272860 | 1.10E-05 | 4.71E-10 |  |  |
| ***NSL1*** | chr1 | 212726153 | 212791782 | 1.51E-04 | 5.33E-06 |  |  |
| ***INPP5F*** | chr10 | 119726042 | 119829147 | 6.46E-04 | 2.50E-06 |  |  |

**Supplementary Table 3(ST3).** Susceptibility genes identified by the ASTWAS and 3`aTWAS models in the RA dataset.

| Gene | Chr | Start | End | ASTWAS_P | 3`aTWAS_P | Reference | MalaCards |
| --- | --- | --- | --- | --- | --- | --- | --- |
| ***SNHG32*** | chr6 | 31822094 | 31839766 | 7.88E-43 | 1.84E-12 |  |  |
| ***SAPCD1-AS1*** | chr6 | 3106199 | 3107477 | 1.84E-42 | 1.97E-11 |  |  |
| ***PBX2*** | chr6 | 3489722 | 3495164 | 4.00E-42 | 3.52E-43 | PMID:20017998? |  |
| ***GPANK1*** | chr6 | 2917047 | 2922107 | 4.41E-42 | 1.60E-35 | PMID:39789419 |  |
| ***MSH5*** | chr6 | 3217300 | 3242109 | 5.63E-42 | 4.44E-35 |  |  |
| ***ATF6B*** | chr6 | 3457284 | 3470273 | 2.15E-41 | 6.08E-44 | PMID:38835753 |  |
| ***CSNK2B*** | chr6 | 2970283 | 2977329 | 5.10E-41 | 1.32E-37 |  |  |
| ***CLIC1*** | chr6 | 31730581 | 31739763 | 6.22E-41 | 1.60E-35 | PMID:34410943 |  |
| ***PRRC2A*** | chr6 | 31620715 | 31637771 | 1.17E-40 | 4.21E-02 |  | TRUE |
| ***NELFE*** | chr6 | 3294123 | 3301128 | 6.96E-40 | 3.43E-26 | PMID:39789419 |  |
| ***CYP21A1P*** | chr6 | 3306878 | 3309588 | 1.11E-39 | 1.60E-35 |  |  |
| ***NOTCH4*** | chr6 | 3418042 | 3447269 | 1.40E-39 | 1.60E-35 | PMID23318300 |  |
| ***LOC100507547*** | chr6 | 32152802 | 32154365 | 3.61E-39 | 6.33E-35 |  |  |
| ***HLA-DRA*** | chr6 | 3662891 | 3668101 | 5.50E-39 | 2.72E-10 |  |  |
| ***RNF5*** | chr6 | 3483335 | 3485774 | 8.28E-39 | 4.21E-02 | 10.1002/rai2.12145 | |
| ***HLA-B*** | chr6 | 31353872 | 31367067 | 3.11E-38 | 4.06E-17 |  | TRUE |
| ***C4A*** | chr6 | 3374766 | 3378295 | 2.21E-37 | 7.23E-13 |  |  |
| ***CYP21A2*** | chr6 | 3380335 | 3383703 | 3.34E-37 | 4.34E-07 | 10.4078/jrd.2019.26.2.131? | |
| ***PRRT1*** | chr6 | 32148359 | 32153083 | 1.40E-36 | 4.14E-32 |  |  |
| ***DDAH2*** | chr6 | 3032085 | 3035664 | 1.06E-35 | 6.85E-23 |  |  |
| ***HLA-DRB5*** | chr6 | 32517353 | 32530287 | 3.77E-35 | 8.63E-09 |  |  |
| ***C4B*** | chr6 | 3324061 | 3324087 | 6.86E-35 | 1.83E-07 | PMID:22076784 |  |
| ***LST1*** | chr6 | 2891165 | 2893950 | 6.87E-31 | 3.43E-26 |  |  |
| ***HLA-DRB1*** | chr6 | 32577902 | 32589848 | 8.92E-27 | 1.39E-17 | PMID:32370106 | TRUE |
| ***STK19*** | chr6 | 31971091 | 31982821 | 2.92E-26 | 2.38E-19 |  |  |
| ***SKIV2L*** | chr6 | 31959116 | 31969818 | 9.74E-22 | 3.22E-15 |  |  |
| ***DXO*** | chr6 | 3311846 | 3314328 | 6.14E-21 | 4.44E-02 |  |  |
| ***HLA-DPA1*** | chr6 | 4363634 | 4379828 | 1.56E-19 | 5.04E-10 | 10.21203/rs.3.rs-700226/v1 | |
| ***HLA-DRB6*** | chr6 | 32553046 | 32558186 | 2.42E-19 | 9.17E-24 |  |  |
| ***BRD2*** | chr6 | 32968594 | 32981505 | 3.85E-17 | 1.08E-09 |  |  |
| ***HLA-DQB1*** | chr6 | 4076917 | 4086211 | 8.86E-17 | 5.20E-08 | PMID:28455285 | TRUE |
| ***PSMB9*** | chr6 | 32844136 | 32860734 | 5.47E-16 | 9.29E-13 | PMID:36045515 |  |
| ***HLA-DMA*** | chr6 | 4142920 | 4165041 | 3.16E-14 | 2.99E-05 | PMID:27898717 | TRUE |
| ***HLA-DPB1*** | chr6 | 33075936 | 33089696 | 1.37E-12 | 1.41E-08 | PMID:29425827 |  |
| ***PHF1*** | chr6 | 4604785 | 4605630 | 3.14E-11 | 7.10E-07 |  |  |
| ***LOC100294145*** | chr6 | 32894144 | 32903758 | 9.75E-11 | 1.61E-18 |  |  |
| ***TAPBP*** | chr6 | 33299694 | 33314284 | 1.40E-10 | 2.25E-04 | PMID:27445359 |  |
| ***RGL2*** | chr6 | 4727590 | 4735260 | 1.47E-10 | 2.98E-02 | PMID: 37644603? |  |
| ***PHTF1*** | chr1 | 113696831 | 113759489 | 2.42E-10 | 2.44E-08 | PMID:33740106 |  |
| ***HLA-DQA1*** | chr6 | 3836385 | 3842664 | 3.51E-10 | 3.12E-07 | PMID:30936065 |  |
| ***AP4B1-AS1*** | chr1 | 113812379 | 113901237 | 1.65E-09 | 7.58E-08 |  |  |
| ***SLC39A7*** | chr6 | 33200305 | 33204439 | 3.34E-09 | 3.95E-03 | PMID:20018006? |  |
| ***NUP98*** | chr11 | 3671083 | 3797792 | 3.66E-09 | 5.32E-03 |  |  |
| ***SYNGAP1*** | chr6 | 33436829 | 33454470 | 1.17E-08 | 8.23E-07 |  |  |
| ***RSBN1*** | chr1 | 113761832 | 113812476 | 2.10E-08 | 4.91E-02 | PMID:33740106 |  |
| ***PWP1*** | chr12 | 107685799 | 107713162 | 3.40E-08 | 4.34E-09 |  |  |
| ***NEU1*** | chr6 | 31857063 | 31862905 | 5.34E-08 | 3.34E-08 |  |  |
| ***MICA*** | chr6 | 31382711 | 31400793 | 6.03E-08 | 5.76E-05 | PMID:19409079 |  |
| ***TAP2*** | chr6 | 32821833 | 32838739 | 8.75E-08 | 1.75E-04 | PMID:24972609 |  |
| ***NFKBIL1*** | chr6 | 31546870 | 31558829 | 1.52E-07 | 9.28E-05 |  | TRUE |
| ***TCF19*** | chr6 | 31158331 | 31167159 | 2.57E-07 | 5.76E-05 |  |  |
| ***DDR1*** | chr6 | 2346729 | 2355691 | 7.31E-07 | 5.71E-04 |  |  |
| ***PSMB8-AS1*** | chr6 | 4263439 | 4265848 | 8.16E-07 | 6.66E-02 |  |  |
| ***HLA-C*** | chr6 | 2577801 | 2581172 | 8.38E-07 | 9.14E-09 | PMID:23901134 |  |
| ***HLA-DQB2*** | chr6 | 3950399 | 3957838 | 2.32E-06 | 1.11E-06 |  |  |
| ***TUBB*** | chr6 | 2030452 | 2035677 | 3.69E-06 | 2.73E-06 |  |  |
| ***HLA-J*** | chr6 | 1304413 | 1307346 | 9.45E-06 | 8.48E-07 |  |  |
| ***PRDX6*** | chr1 | 173477330 | 173488815 | 1.07E-03 | 2.32E-06 |  |  |
| ***GBP5*** | chr1 | 89256189 | 89272860 | 1.54E-02 | 2.62E-07 | PMID:33159795 |  |


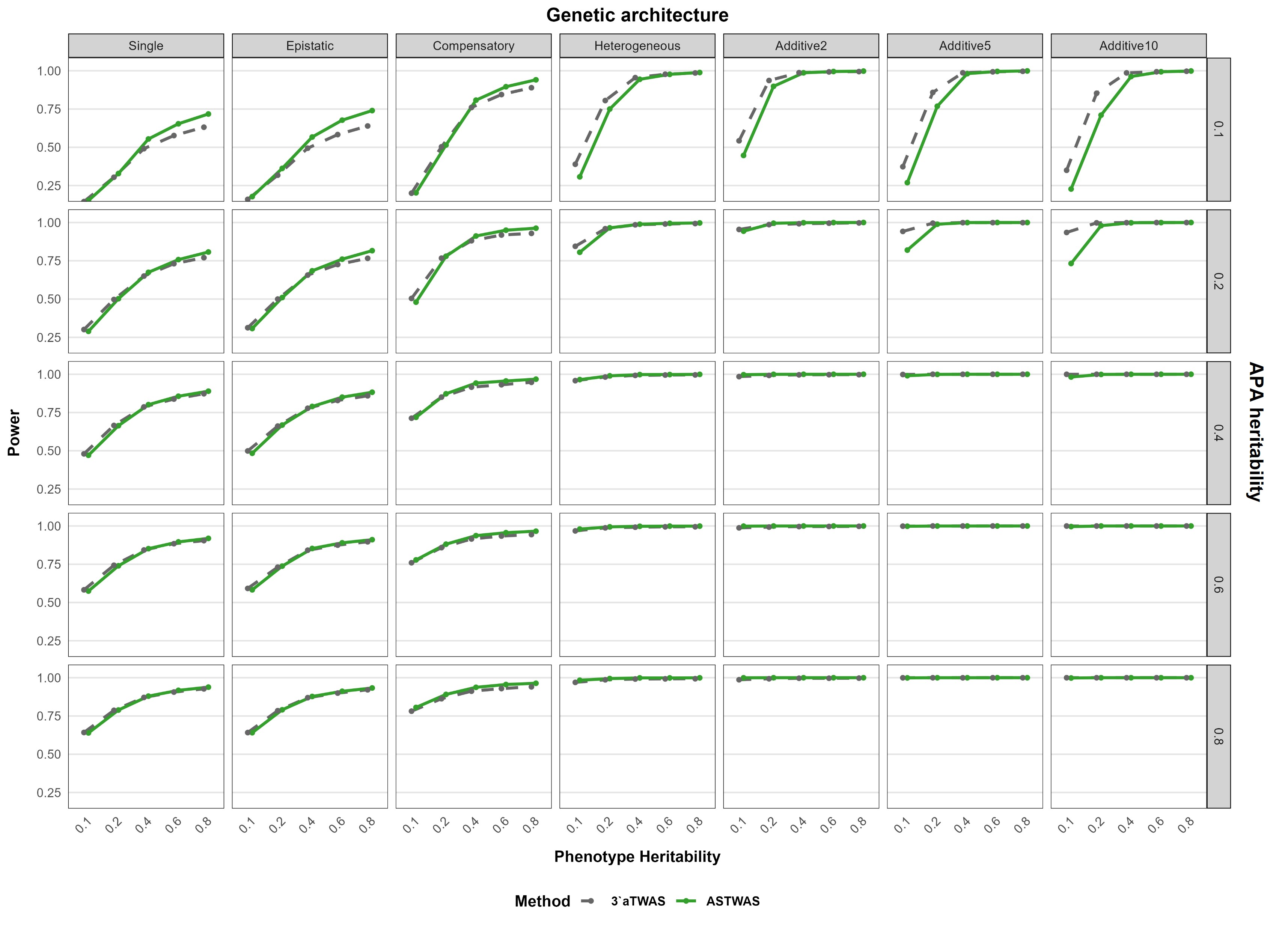


**Supplementary Figure 1(SF1).** Statistical power of ASTWAS and 3`aTWAS models under the causal genetic assumption with expanded heritability scenarios. This simulation was conducted in response to the reviewers' comments on the model's robustness under more genetic coefficient scenarios. The x-axis represents the phenotypic heritability, the left y-axis represents the statistical power of the model, and the right y-axis represents the APA heritability.

**Alt text:** Multi-panel line graphs illustrating the statistical power of ASTWAS and 3'aTWAS models under a causal genetic assumption. The grid displays power trends across varying phenotypic heritabilities (x-axis), APA heritabilities (rows), and genetic architectures (columns). The green solid line representing ASTWAS generally indicates higher power compared to the grey dashed line for 3'aTWAS, particularly within single, epistatic and compensatory architectures, whilst both models approach maximum power in additive scenarios.


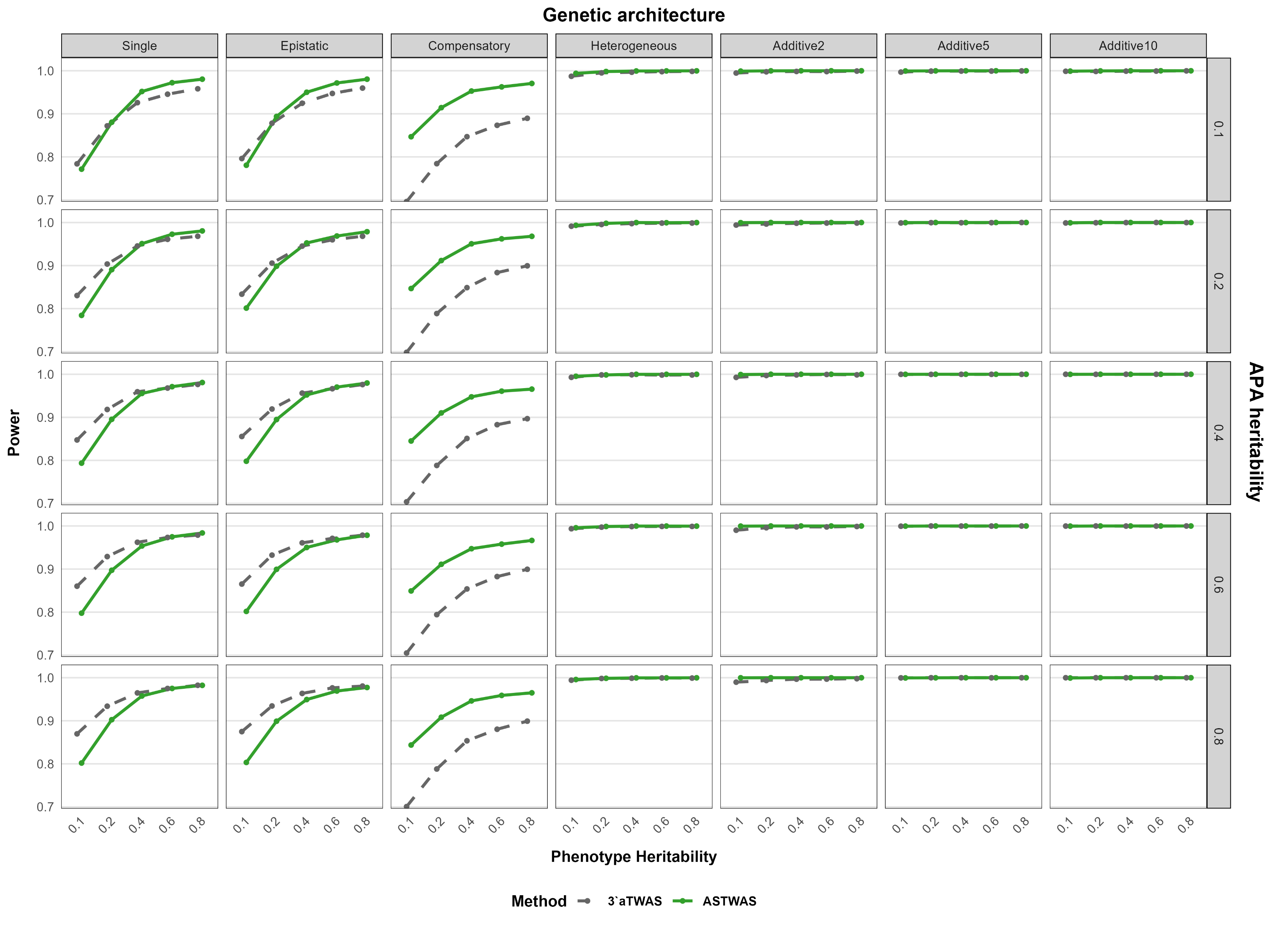


**Supplementary Figure 2(SF2).** Statistical power of ASTWAS and 3`aTWAS models under the pleiotropy assumption with expanded heritability scenarios. This simulation was conducted in response to the reviewers' comments on the model's robustness under more genetic coefficient scenarios. The x-axis represents the phenotypic heritability, the left y-axis represents the statistical power of the model, and the right y-axis represents the APA heritability.

**Alt text:** Multi-panel line graphs comparing the statistical power of ASTWAS and 3'aTWAS models under a pleiotropy assumption. Plots are arranged by genetic architecture (columns) and APA heritability levels (rows), with phenotype heritability on the x-axis. The data suggests that ASTWAS, depicted by a green solid line, maintains a power advantage over the 3'aTWAS model (grey dashed line) across most conditions, showing increasing power as heritability rises.


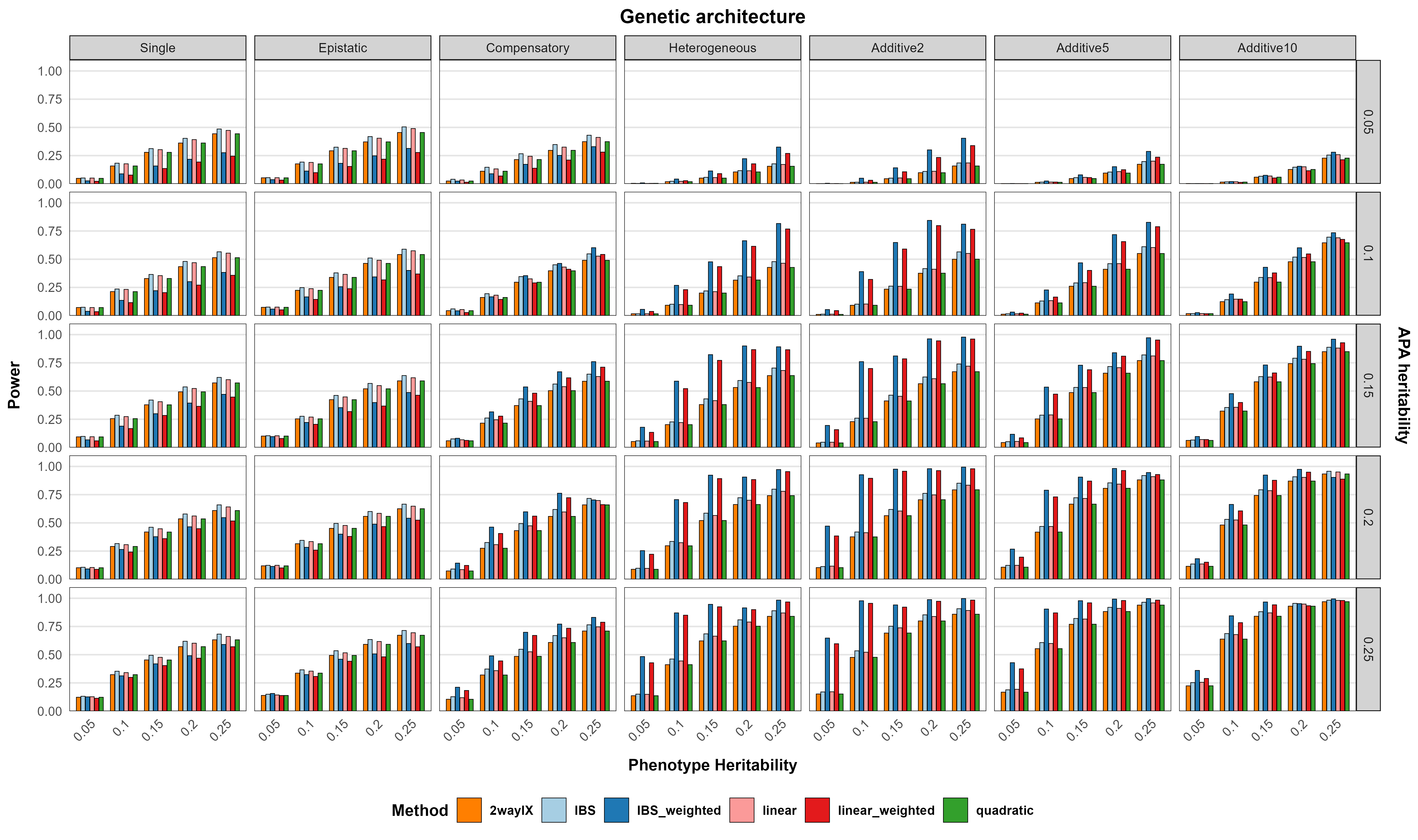


**Supplementary Figure 3(SF3).** Statistical power comparison of different kernel functions within the ASTWAS framework under the causal genetic assumption. This simulation was conducted in response to the reviewers' comments regarding the impact of kernel selection on the model's power. The x-axis represents the phenotypic heritability, the left y-axis represents the statistical power, and the right y-axis represents the APA heritability. This analysis was conducted in response to reviewer comments to evaluate the robustness of the ASTWAS method to kernel choice.

**Alt text:** Multi-panel bar charts comparing the statistical power of six kernel functions (including linear, quadratic, IBS, and their weighted variants) within the ASTWAS framework under a causal genetic assumption. Panels are stratified by genetic architecture (columns) and APA heritability (rows), with phenotypic heritability on the x-axis. The analysis indicates that weighted kernels, particularly IBS_weighted and linear_weighted, generally demonstrate robust power trends across varying heritability scenarios compared to unweighted alternatives.


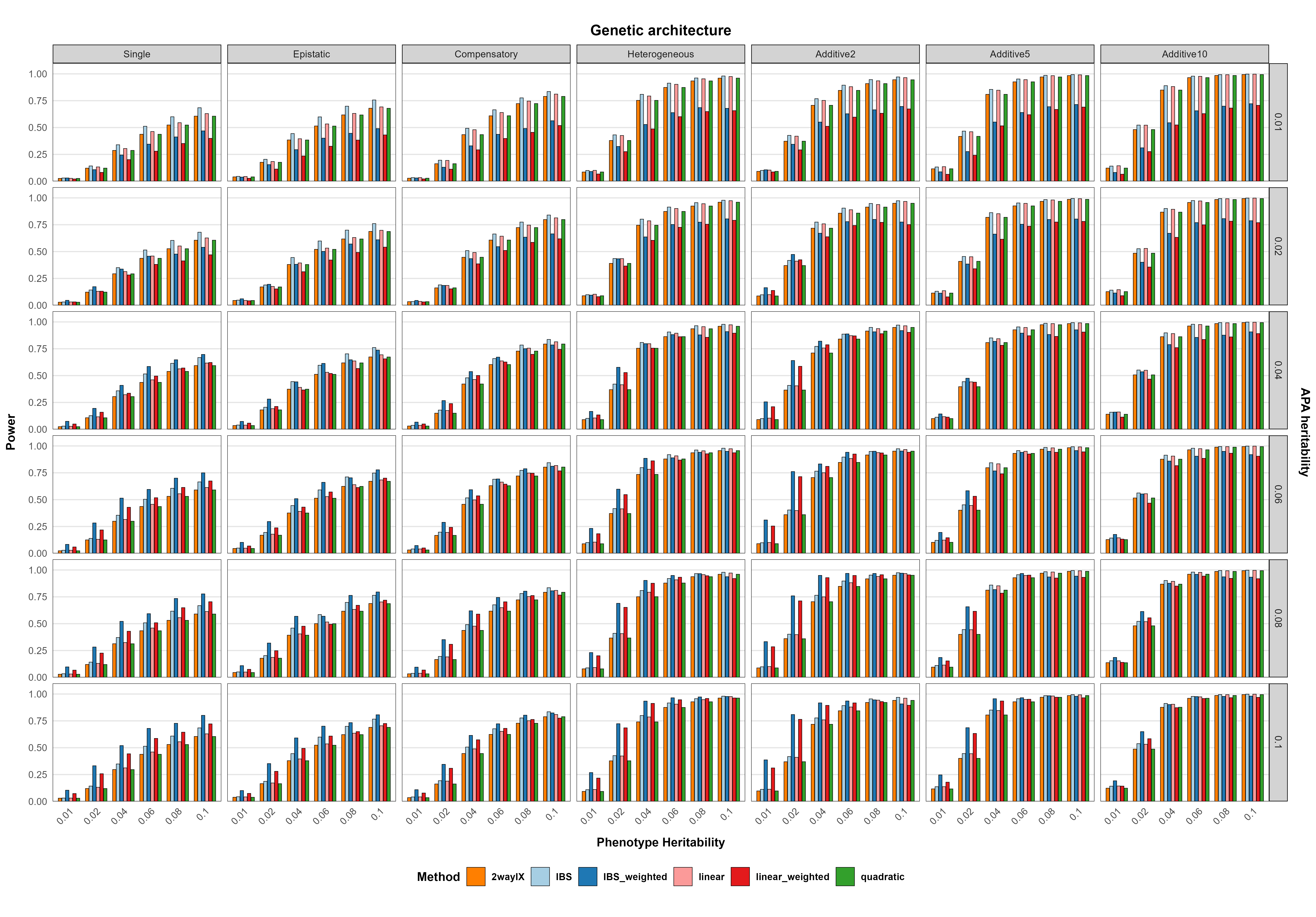


**Supplementary Figure 4(SF4).** Statistical power comparison of different kernel functions within the ASTWAS framework under the pleiotropy assumption. This simulation was conducted in response to the reviewers' comments regarding the impact of kernel selection on the model's power. The x-axis represents the phenotypic heritability, the left y-axis represents the statistical power, and the right y-axis represents the APA heritability. This analysis was conducted in response to reviewer comments to evaluate the robustness of the ASTWAS method to kernel choice.

**Alt text:** Multi-panel bar charts illustrating the statistical power of different kernel functions under a pleiotropy assumption, arranged by genetic architecture and APA heritability. The x-axis denotes phenotypic heritability. The data suggests that whilst power generally increases with heritability across all kernels, weighted functions such as linear_weighted and IBS_weighted frequently exhibit competitive performance compared to non-weighted alternatives like 2wayIX or quadratic across complex genetic architectures.


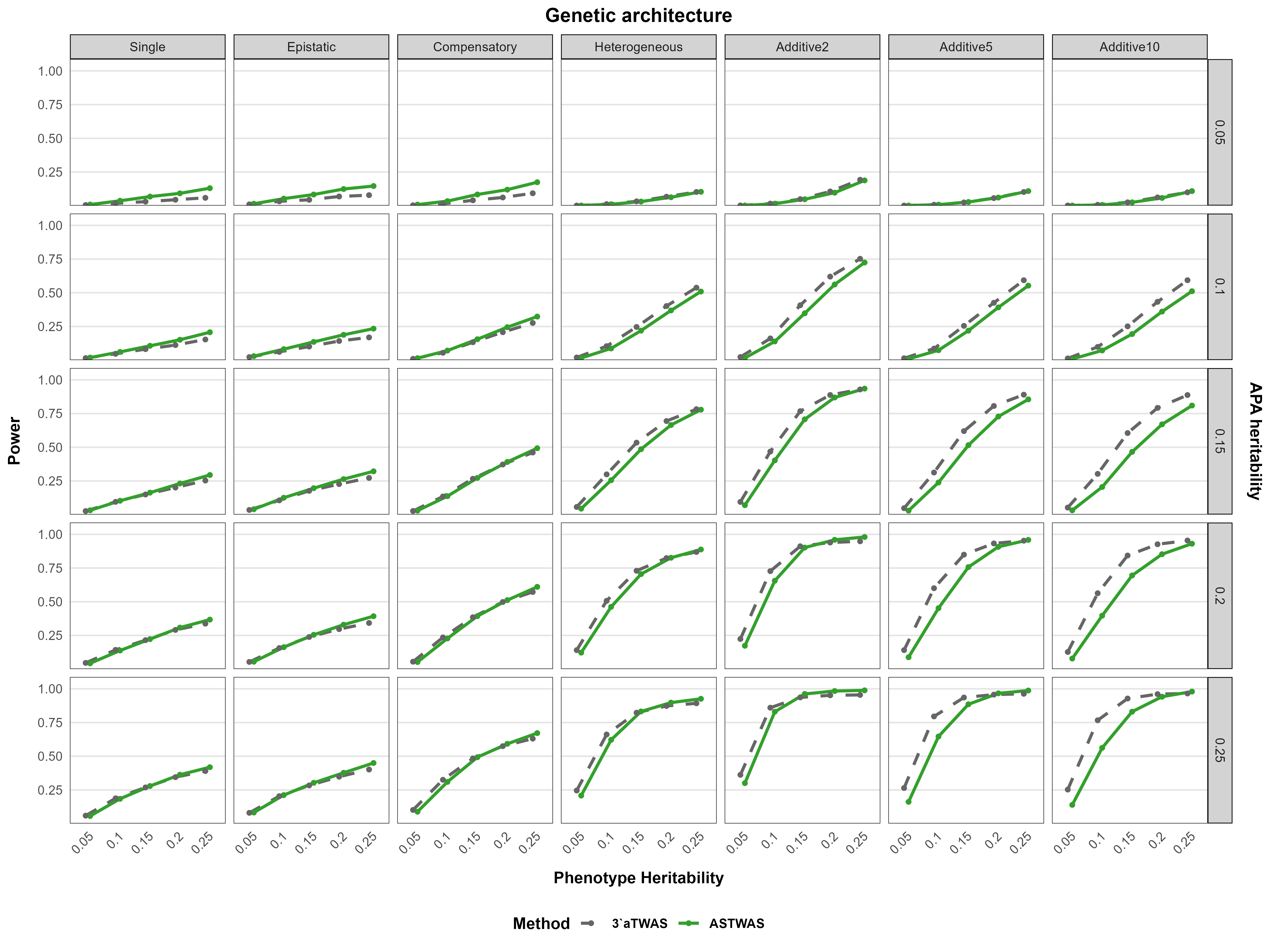


**Supplementary Figure 5(SF5).** Statistical power of ASTWAS and 3`aTWAS models under causal genetic assumptions for binary (case-control) traits. This simulation was conducted in response to the reviewers' comments regarding the statistical power of the model on binary simulated trait data. The x-axis represents the phenotypic heritabilit, the left y-axis represents the statistical power, and the right y-axis represents the APA heritability. This experiment selected seven genetic structures (columns), five phenotypic heritability values (x-axis: 0.05, 0.1, 0.15, 0.2, 0.25), and five APA heritability values (rows: 0.05, 0.1, 0.15, 0.2, 0.25) to simulate 175 sets of data. The line chart provides a comprehensive evaluation of the statistical power of 3`aTWAS and ASTWAS under these binary trait conditions.

**Alt text:** Multi-panel line graphs illustrating the statistical power of ASTWAS and 3'aTWAS models for binary traits under causal genetic assumptions. Panels are arranged by genetic architecture (columns) and APA heritability (rows), plotting power against phenotypic heritability (x-axis). The green solid line representing ASTWAS generally indicates higher or comparable power to the 3'aTWAS model (grey dashed line), particularly in single, epistatic and compensatory architectures, with power increasing alongside phenotypic heritability.


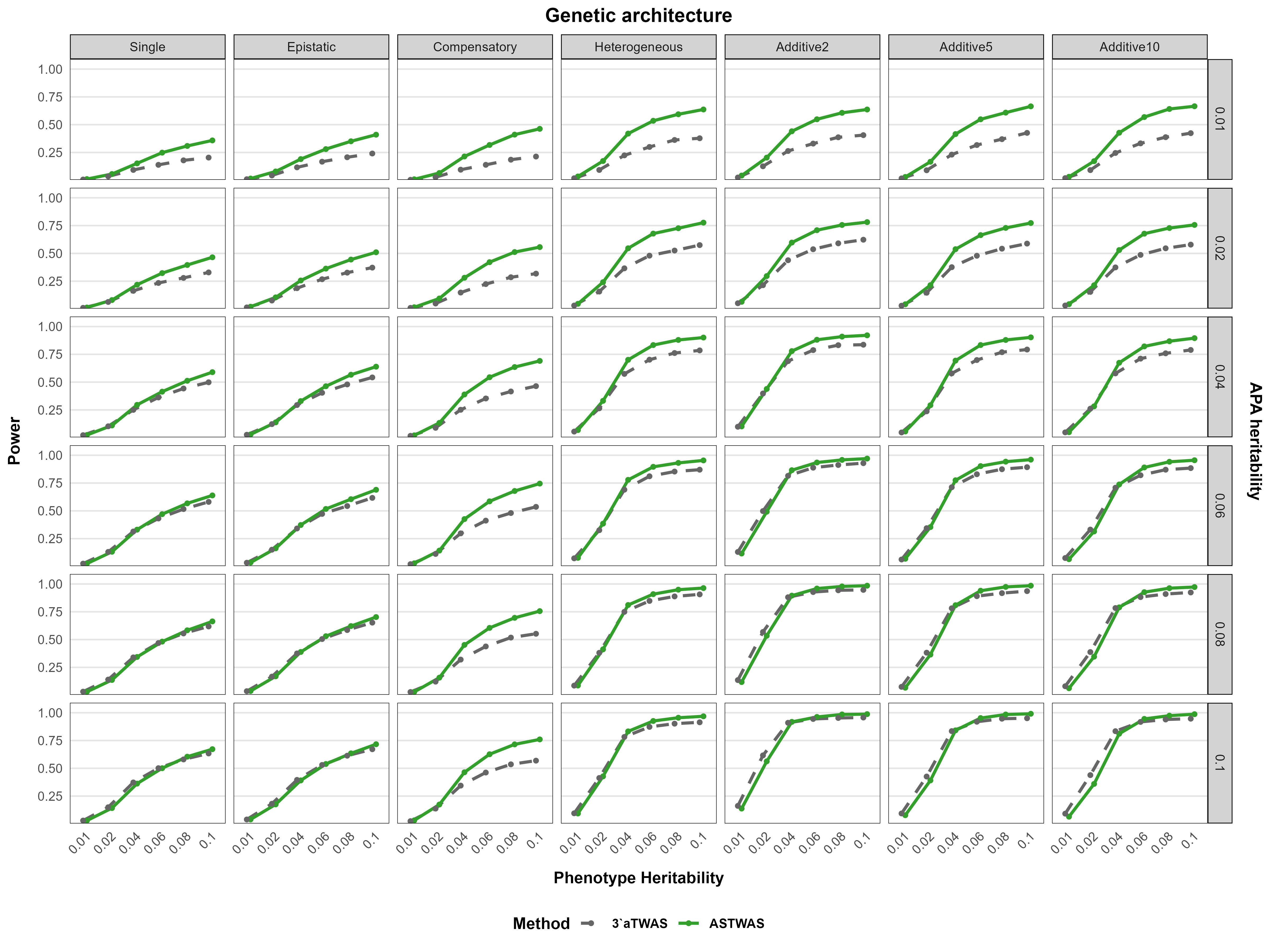


**Supplementary Figure 6(SF6).** Statistical power of ASTWAS and 3`aTWAS models under pleiotropic assumptions for binary (case-control) traits. This simulation was conducted in response to the reviewers' comments regarding the statistical power of the model on binary simulated trait data. The x-axis represents the phenotypic heritability, the left y-axis represents the statistical power, and the right y-axis represents the APA heritability. This experiment selected seven genetic structures (columns), six phenotypic heritability values (x-axis: 0.01, 0.02, 0.04, 0.06, 0.08, 0.10), and six APA heritability values (rows: 0.01, 0.02, 0.04, 0.06, 0.08, 0.10) to simulate 252 sets of data. The line chart provides a comprehensive evaluation of the statistical power of 3‘aTWAS and ASTWAS under these binary trait conditions.

**Alt text:** Multi-panel line graphs displaying statistical power comparisons for binary traits under pleiotropic assumptions. Stratified by genetic architecture and APA heritability, the plots show power trends against phenotypic heritability on the x-axis. The data suggests that ASTWAS (green solid line) consistently outperforms or matches 3'aTWAS (grey dashed line), exhibiting robust power advantages in complex genetic scenarios such as compensatory and heterogeneous architectures across varying heritability levels.


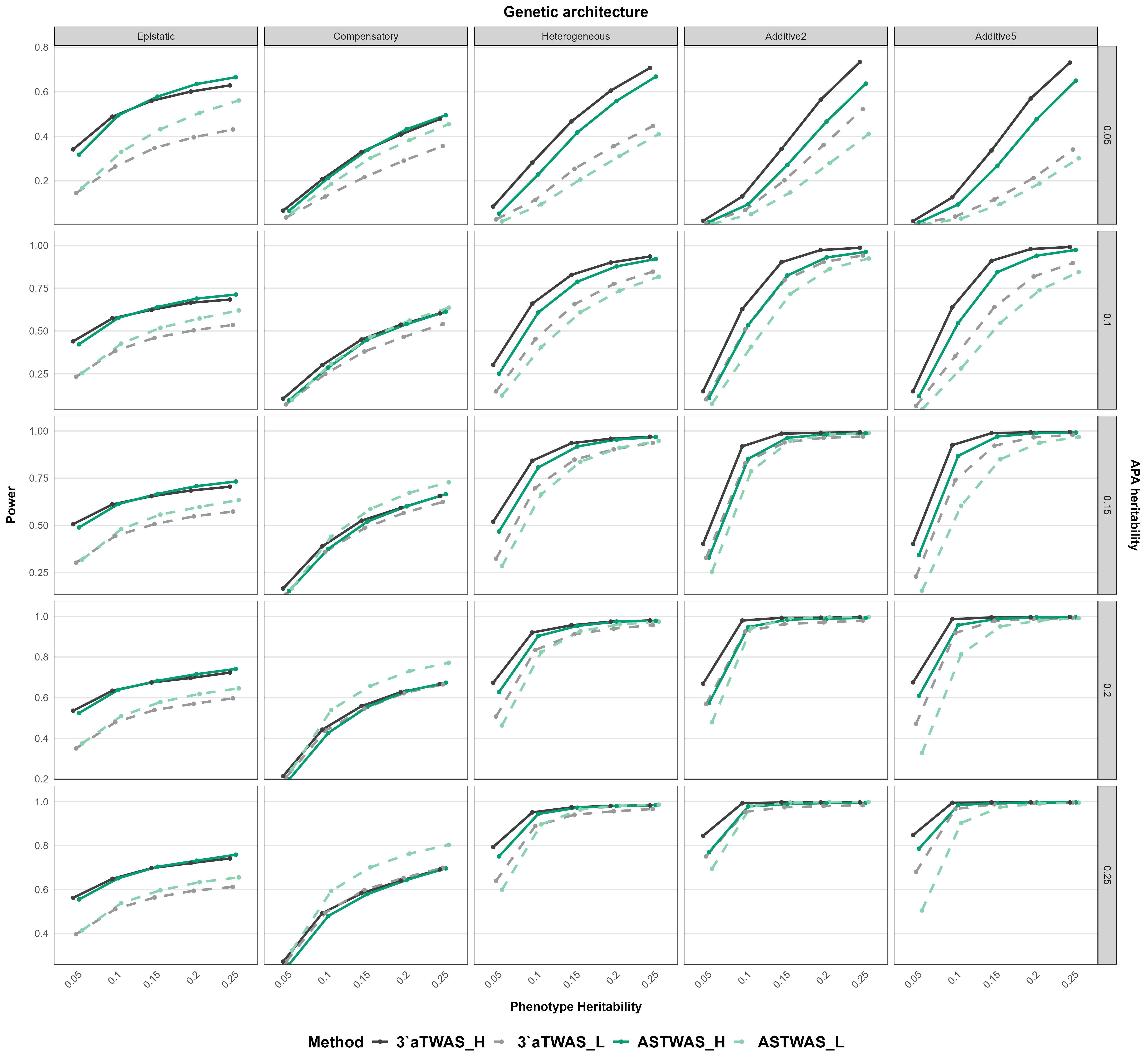


**Supplementary Figure 7(SF7).** Statistical power comparison of 3'aTWAS and ASTWAS models under causal genetic assumptions considering different Linkage Disequilibrium (LD) structures. This simulation was conducted in response to reviewer comments regarding LD structure. Causal variants were selected based on two scenarios: High LD (LD coefficient r^2^ > 0.8, denoted by _H in the legend) and Low LD (LD coefficient r^2^ < 0.2, denoted by _L in the legend). The x-axis represents phenotypic heritability, the left y-axis represents statistical power, and the right y-axis represents APA heritability. This experiment selected five genetic structures (columns, excluding Single and Additive10), five phenotypic heritability values (x-axis: 0.05, 0.1, 0.15, 0.2, 0.25), and five APA heritability values (rows: 0.05, 0.1, 0.15, 0.2, 0.25). The line chart evaluates the robustness of both models to different LD patterns.

**Alt text:** Multi-panel line charts comparing statistical power of ASTWAS and 3'aTWAS under causal genetic assumptions, stratified by high and low linkage disequilibrium (LD). Panels display power against phenotypic heritability across varied genetic architectures and APA heritability levels. Data indicates that ASTWAS generally exhibits superior power compared to 3'aTWAS, with high LD scenarios (solid lines) consistently yielding higher power than low LD scenarios (dashed lines).


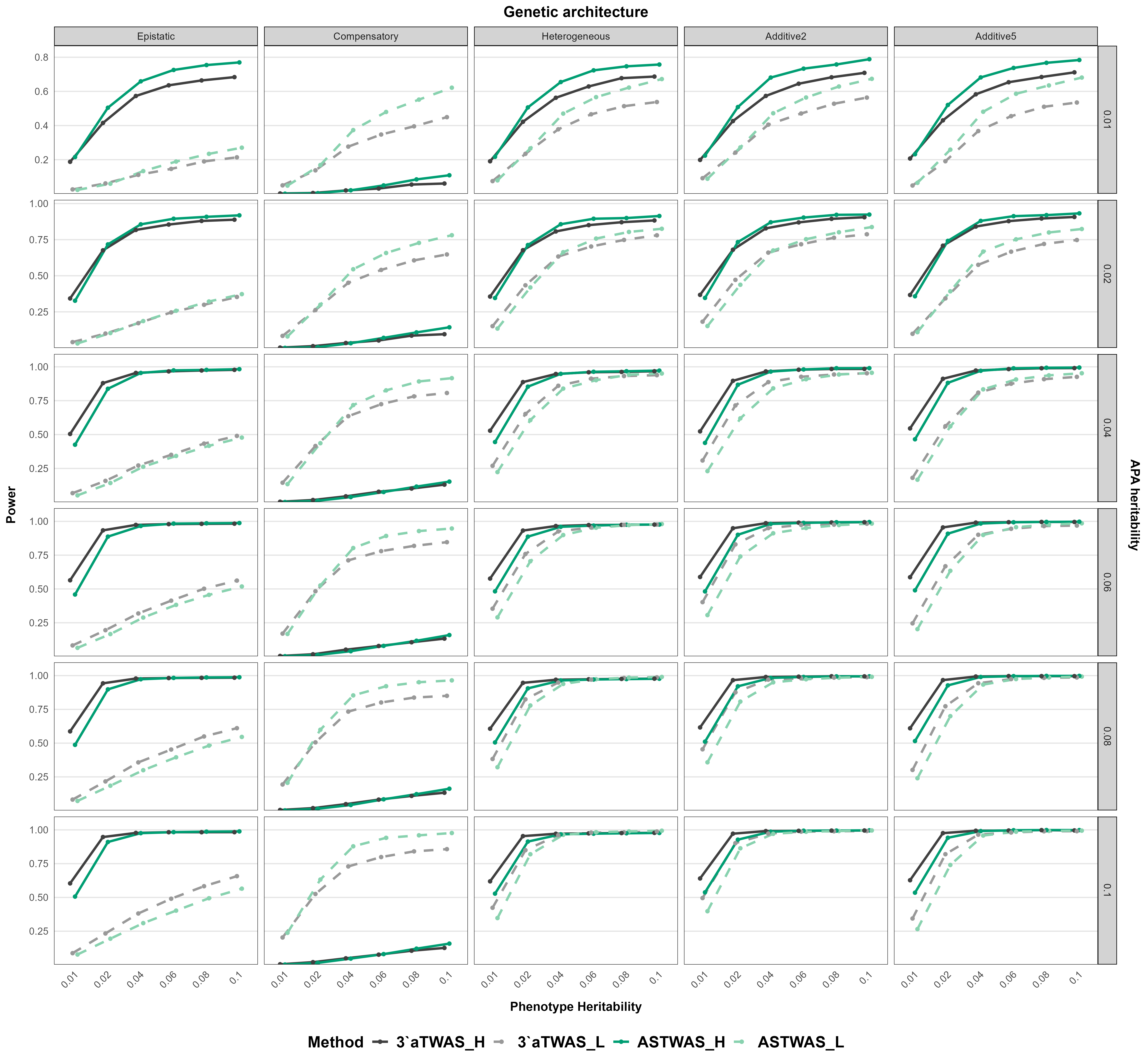


**Supplementary Figure 8(SF8).** Statistical power comparison of 3'aTWAS and ASTWAS models under pleiotropic assumptions considering different Linkage Disequilibrium (LD) structures. This simulation was conducted in response to reviewer comments regarding LD structure. Causal variants were selected based on two scenarios: High LD (LD coefficient r^2^ > 0.8, denoted by _H in the legend) and Low LD (LD coefficient r^2^ < 0.2, denoted by _L in the legend). The x-axis represents phenotypic heritability, the left y-axis represents statistical power, and the right y-axis represents APA heritability. This experiment selected five genetic structures (columns, excluding Single and Additive10), six phenotypic heritability values (x-axis: 0.01, 0.02, 0.04, 0.06, 0.08, 0.1), and six APA heritability values (rows: 0.01, 0.02, 0.04, 0.06, 0.08, 0.1). The line chart evaluates the robustness of both models to different LD patterns.

**Alt text:** Multi-panel line charts illustrating the statistical power of ASTWAS and 3'aTWAS under pleiotropic assumptions, accounting for high and low linkage disequilibrium. Plots denote power relative to phenotypic heritability across five genetic architectures. The trends suggest that ASTWAS (green lines) maintains robust performance advantages over 3'aTWAS (grey lines), whilst models utilising high LD variants (solid lines) demonstrate greater power than those using low LD variants (dashed lines).
